# Quantitative evaluation of quality indicators and flavor characteristics of the same variety of chili from different producing areas of China: An integrated multivariate analysis

**DOI:** 10.1016/j.fochx.2026.104589

**Published:** 2026-10-07

**Authors:** Wenxin Li, Yong Yin, Peng Zhou, Jia Yan, Yongping Wang, Xiaojing Sun, Ju Chen, Min Lu, Xueya Wang, Jianwen He

**Affiliations:** a Chili Pepper Research Institute, Guizhou Academy of Agricultural Science, Guiyang, Guizhou 550006, China; b College of Artificial Intelligence, Southwest University, Chongqing 400715, China

**Keywords:** Chili pepper, Meteorological factors, Producing areas, Untargeted metabolomics, Volatile flavor compounds

## Abstract

This study investigated the effects of different producing areas on the quality of the chili pepper variety Layan 12, encompassing chemical properties, volatile flavor components, untargeted metabolites, and meteorological factors. Significant quality variations were observed among the four regions. A total of 103 volatile flavor compounds were identified, of which 11 were classified as differential compounds. Notably, concentrations of beta-cyclocitral and dihydroactinidiolide were higher in samples from Guizhou. Untargeted metabolomics revealed 3419 metabolites, among which 5 were annotated as key characteristic components. Correlation analysis indicated that global radiation, relative humidity, temperature, and diurnal temperature range are important environmental factors associated with chili fruit quality formation. Principal component analysis demonstrated that Guizhou achieved the highest comprehensive score, followed by Henan and Inner Mongolia. These findings provide a scientific basis for the selection of optimal chili producing areas.

## Highlights

• Chili peppers from different producing areas exhibit significant variations in chemical indicators, flavor compounds, and untargeted metabolites.
• Layan 12 contained nine categories of volatile flavor compounds, with esters being the most abundant.
• Layan 12 contained 3419 types, 16 classes of untargeted metabolites.
• Global radiation, relative humidity, temperature, and diurnal temperature range are important environmental factors associated with the formation of chili fruit quality.
• Principal component analysis integrating chemical indicators, flavor compounds, and utargeted metabolites demonstrated that the Layan 12 variety cultivated in Guizhou achieved the highest overall score.

## 1 Introduction

Chili pepper is one of the most widely consumed flavoring crops worldwide (Li, Chen, et al., 2023). The total global production of chili pepper reached 41.88 million tons in 2022, with China ranking first at 17.16 million tons (FAO; www.fao.org). Guizhou and Henan Provinces have relatively large chili pepper cultivation areas in China. Moreover, cultivation areas in sparsely populated regions such as Xinjiang and Inner Mongolia have been expanding annually, driven by labor productivity costs and the increasing demand for automated mechanical production (Zou et al., 2025). In addition to fresh consumption, chili peppers are products such as dried chili, fried chili sauce, and fermented chili (Hui et al., 2023; Song et al., 2022). Their extracts are also widely used in the pharmaceutical, feed, and cosmetic industries (Mi et al., 2022; Vittorazzi et al., 2022). The area dedicated to processed chili accounts for approximately 50% of the total chili cultivation area (Mi et al., 2022; Vittorazzi et al., 2022; Zou et al., 2025). Different processed products require distinct quality attributes of chili. For instance, a previous study (Wang et al., 2025) investigated the processing adaptability of Zao Chili reporting that varieties with high reducing sugar content and low crude fiber content are more suitable for fermented chili production. Chili quality is closely related to the producing area in addition to the variety. Previous studies indicate that variations in environmental factors at production sites (including altitude, latitude, longitude, climate, temperature, humidity, light, and soil), as well as differences in field management practices, influence the secondary metabolic processes of chili pepper, thereby affecting its physicochemical properties, flavor compounds, and metabolite content (Li et al., 2025, Liang et al., 2024, Yang et al., 2024, Yu et al., 2026). The selection of chili raw materials for fermented Pixian Douban should account for both origin and variety (Wang et al., 2020). Volatile substances of landmark chili varieties differed between landmark and non-landmark areas (Chen et al., 2023). Therefore, elucidating the relationship between chili quality and producing areas is essential for leveraging the advantages of specific production regions. Through the research on the physicochemical indicators and flavor compound differences of fried pepper sauce prepared from chili peppers from five production areas, it was found that the production area has a certain influence on the quality of fried chili sauce (Yang et al., 2023).

Headspace solid-phase microextraction-gas chromatography–mass spectrometry (HS-SPME-GC–MS) is widely used as an effective method for extracting volatile substances (Xi et al., 2024), with applications in wine, tea and fermented food, among others (Bai et al., 2025, Hui, Fang, Ma, Hamid and Li, 2023, Pu et al., 2023, Xu et al., 2021). Metabolomics uses high-throughput analytical technique for the comprehensive qualitative and quantitative analysis of metabolites (Li et al., 2021), In recent years, metabolomics has been widely applied to quality control, variety improvement and process optimization (Mattoli et al., 2023; Wang et al., 2022; Wang & Li, 2024).

This study analyzed the chemical properties, volatile flavor, and untargeted metabolic characteristics of the same chili variety from different producing areas. Using as indicators: (1) eight chemical indexes (reducing sugar, fat, protein, crude fiber, total amino acid, capsaicin, dihydrocapsaicin and capsanthin); (2) differential volatile flavor substances determined by odor activity value (OAV), principal component analysis (PCA) and orthogonal partial least squares discriminant analysis (OPLS-DA); and (3) key characteristic components obtained by untargeted metabolism analysis as indicators, comprehensive evaluation on Layan 12 from different producing areas was performed using principal component analysis, so as to explore the effects of different origins on its quality. In particular, this study establishes a quantifiable quality evaluation index system for chili production areas, integrating physicochemical indices, volatile flavor compounds and key metabolites into a comprehensive scoring model, which provides an objective and reproducible approach for comparing and selecting optimal production regions. This unique contribution distinguishes the present work from previous studies that largely relied on qualitative comparisons of individual quality attributes. The purpose of this study was to provide theoretical guidance and technical support for improving chili quality.

## 2 Materials and methods

### 2.1 Materials

Chili pepper variety Layan 12 was harvested in September 2023 from four production sites: Xinjiang (B2, Urumqi, Xinjiang; 43.96°N, 87.50°E; altitude 593 m), Inner Mongolia (B6, Chifeng, Inner Mongolia; 42.37°N, 118.82°E; altitude 610 m), Henan (B7, Zhengzhou, Henan; 34.82°N, 113.98°E; altitude 86 m) and Guizhou (B8, Guiyang, Guizhou; 26.51°N, 106.67°E; altitude 1147 m).

### 2.2 Acquisition of meteorological data

Meteorological data were obtained from the Xihe Energy Meteorological Big Data Platform. Six meteorological factors covering the entire growth period of chili plants from planting to harvest (May–September) were analyzed: maximum temperature, diurnal temperature range, effective accumulated temperature ≥ 10 °C, average relative humidity, total radiation, and the ratio of diffuse radiation. Among these factors, total radiation and effective accumulated temperature ≥ 10 °C were calculated as cumulative values, while the ratio of diffuse radiation was determined as the ratio of cumulative diffuse radiation to cumulative total radiation. The remaining indicators were calculated as mean values.

### 2.3 Sample preparation

A consistent ridging and double-row planting pattern (plant spacing: 45 cm; row spacing: 60 cm) was adopted across all sites. Field management practices, including water and fertilizer management as well as pest and disease control, were uniformly implemented in accordance with the Guizhou Provincial Standards DB52/T 957 (2014) and DB52/T 958 (2014). At harvest, 15 plants exhibiting uniform growth were selected from each location. A mixed sampling method was employed to collect market-grade fruits from the second to fourth tiers, ensuring that they were free of pests and diseases and of uniform size. The samples were dried in an oven at 60 °C, crushed, sieved, quick-frozen in liquid nitrogen, and stored at −80 °C for subsequent analysis. Sulfuric acid, anhydrous ethanol, and petroleum ether (all analytical grade) were purchased from Ltd., 2-octanol, methanol, tetrahydrofuran (all chromatographic grade) were purchased from Sigma Aldrich (Shanghai) Trading Co.

### 2.4 Detection of chemical indexes

Protein content was determined using the Kjeldahl method according to GB 5009.5, (2016). Fat content was determined according to Soxhlet extraction method in GB 5009.6 (2016). Crude fiber content was determined according to GB/T 5009.10 (2003). Reducing sugar was determined by direct titration method with slight modification according to GB 5009.7 (2016). Capsanthin content was determined by ultrasonic extraction method. Capsaicin was determined according to GB/T 21266, (2007).

### 2.5 GC–MS analysis of the volatile composition

The assay method was based on the literature (Li, Wang, et al., 2023), with slight modification. Volatile flavor compounds in chili samples were analyzed using headspace solid-phase microextraction coupled with gas chromatography–mass spectrometry (HS-SPME-GC–MS). Briefly, 1.0 g of each sample was placed in a 20 mL headspace vial, and 20 μL of 2-octanol (internal standard, 1 μg/μL) was added. The vial was equilibrated at 60 °C for 15 min prior to extraction. The SPME fiber was preconditioned at 250 °C for 15 min, and then exposed to the headspace for 30 min for extraction, followed by thermal desorption at 250 °C for 4 min. Separation was performed on a DB-Wax capillary column (30 m × 250 μm × 0.25 μm) using high-purity helium (99.999%) as the carrier gas at a flow rate of 1 mL/min with a septum purge flow of 3 mL/min. The injector was operated in split mode at 250 °C. The oven temperature was programmed from 40 °C (held for 4 min) to 250 °C at 5 °C/min and held for 5 min. Mass spectrometric analysis was conducted using an electron ionization (EI) source at 70 eV with an emission current of 34.6 μA. The ion source, quadrupole, transfer line, and interface temperatures were set to 230, 150, 250, and 250 °C, respectively. Mass spectra were acquired in full-scan mode over a mass range of 20–550 *m*/*z* without solvent delay. Compound identification was performed by automatic searching and manual interpretation against the National Institute of Standards and Technology (NIST) mass spectral library, with a match score greater than 80% as the criterion for identification. Relative quantification of volatile compounds was carried out using the internal standard method, and the relative content of each compound was calculated according to Eq. (1):

(1) $$C_{i}=\frac{A_{i}}{B}\times \frac{m_{B}}{m}$$

(2) $$\mathrm{OAV}=\frac{C_{i}}{C_{\mathrm{io}}}$$

C_i_: concentration of identified substance i (μg/g), OAV: odor activity value, *A*_*i*_: peak area of substance i, *B*: peak area of internal standard, *m*_*B*_: addition amount of internal standard (g). *m*: sample mass (g), C_*io*:_ aroma threshold of the identified substance i.

### 2.6 Detection of untargeted metabolites

For untargeted metabolomics analysis, the method described by (Song et al., 2022) was followed. Briefly, 50 ± 5 mg of sample was weighed into a 2 mL centrifuge tube, and 400 μL of extraction solution (methanol: water =4:1(v:v)) (containing four internal standards, such as L-2- chlorophenylalanine (0.02 mg/mL), etc.) was added, ground for 6 min (−10 °C, 50 Hz), ultrasonic extracted for 30 min (5 °C, 40 KHz) and stood for 30 min at −20 °C. The samples were centrifuged for 15 min (13,000*g*, 4 °C) and the supernatant was collected for analysis on the UHPLC-Exploris 240 system of Thermo Fisher Scientific Ultra Performance Liquid Chromatography-Tandem Fourier Transform Mass Spectrometry. The quality control (QC) sample were the mixture of the supernatant with 20 μL taken from each sample.

### 2.7 Statistical analysis

Three biological replicates were performed for each group, with each replicate consisting of pooled tissue from fifteen plants exhibiting uniform growth. Statistical evaluations, including analysis of variance (ANOVA), Duncan's multiple range test, correlation analysis, and principal component analysis, were conducted using IBM SPSS 25 software, and figures were plotted using Origin 2021 software. PCA and OPLS-DA were performed using SIMCA 14.1 software. Meta-omic analysis was performed using the online platform of Majorbio Cloud Platform (www.majorbio.com) as detailed by (Ren et al., 2022). The raw data were processed using the metabolomics software Progenesis QI (Waters Corporation, Milford, USA) for baseline filtering, peak identification, integration, retention time correction, and peak alignment. The characteristic peaks were then searched and identified using primary and secondary mass spectrometry databases, including Metlin (https://metlin.scripps.edu/), HMDB (http://www.hmdb.ca/), and the Majorbio Bio-Pharm Technology self-built library. The MS mass error was set to less than 1 × 10–5, and matches with a fragment similarity score greater than 30 were considered valid.

## 3 Results and discussion

### 3.1 Chemical index analysis

Chemical indicators of chili have a direct impact on its quality. As shown in Fig. 1, the chemical indexes of chili varied significantly among different producing areas.

**Fig. 1:**
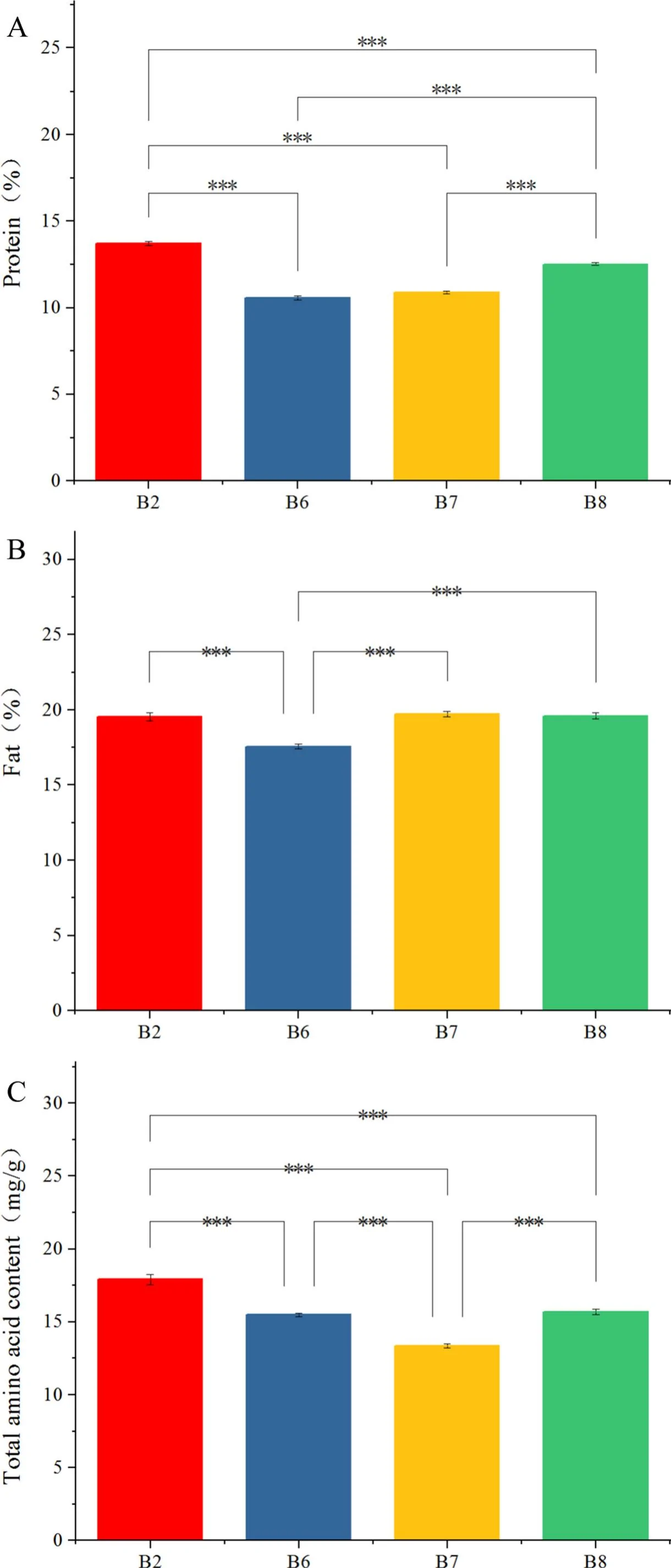

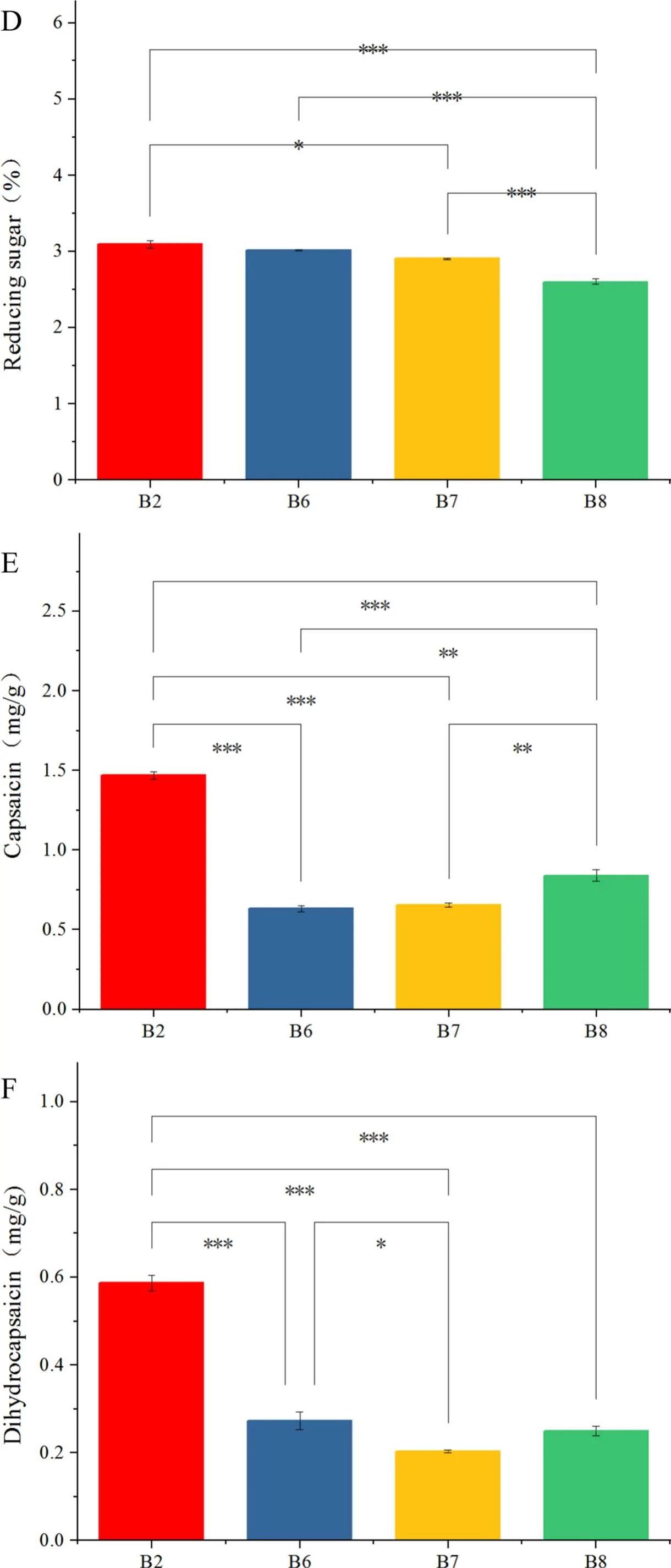

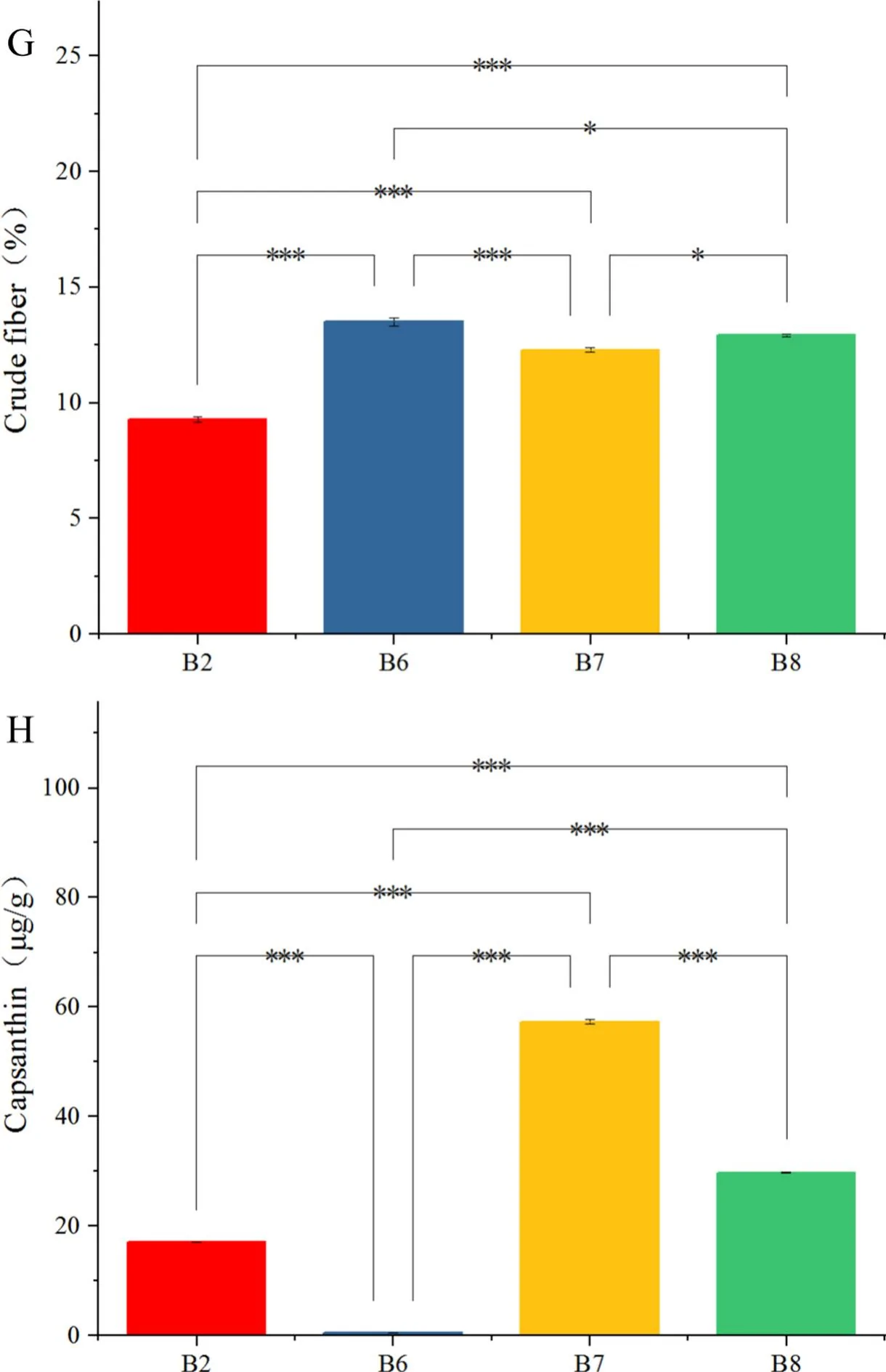
Chemical indexes of Layan 12 grown in different producing areas.(A) Protein, (B) Fat, (C) Total amino acid content, (D) Reducing sugar, (E) Capsaicin, (F) Dihydrocapsaicin, (G) Crude fiber, (H) Capsanthin. Error bars indicate standard error; lowercase letters indicate significant differences. * *p* < 0.05, ** *p* ≤ 0.01, *** *p* ≤ 0.001.

During chili processing, proteins undergo denaturation, leading to the formation of unique flavor compounds that intermingle with the inherent spiciness and aroma, enriching the chili taste (Naik et al., 2022). The protein content of Layan 12 decreased in the order B2 > B8 > B7 > B6, with no significant difference between B6 and B7. Protein and fat affect flavor and texture; fat can act as a lubricant and impart a smoother mouthfeel (Ananthan et al., 2018). The fat content of Layan 12 decreased in the order B6> B8> B7> B2. Additionally, the total amino acid and reducing sugar contents significantly impact the flavor profile of chili. During chili processing, amino acids undergo Maillard reactions with reducing sugars, resulting in the formation of various to produce a range of compounds with potent flavors, including volatiles such as pyrazines and furans (Alonso-Riaño et al., 2024). The total amino acid content of Layan 12 was in the order of B2 > B8 > B6 > B7, and the reducing sugar was in the order of B2 > B6 > B7 > B8. Capsaicinoids and dihydrocapsaicinoids are the main sources of chili spiciness (Wu et al., 2024), the capsaicin of Layan 12 was ranked as B2 > B8 > B7 > B6, and the dihydrocapsaicin was ranked as B2 > B6 > B8 > B7. Chili crude fiber has a great influence on the taste of chili, excessive content would lead to rough texture (Wu et al., 2025), and the crude fiber content of Layan 12 was in the order of B7 > B8 > B2 > B6. Capsaicinoids are alkaloids that produce the pungent sensation and determine the spiciness of chili peppers. In contrast, capsanthin and other carotenoids are the pigments that contribute to the red color of chili pepper products and directly influence their commercial value (Deli et al., 2001), whose content directly determines the redness and commercial value of chili products, the capsanthin of Layan 12 was ranked as B7 > B8 > B2 > B6. In conclusion, B2 of Layan 12 has high capsaicin and dihydrocapsaicin, which is good for extracting capsaicin for deep processing, and B8 has good performance in protein, fat, total amino acid and capsaicin, which is suitable for the preparation of oil chili and extraction of pigment.

### 3.2 Volatile flavor analysis

As shown in Fig. 2, 103 volatile flavor compounds were detected, with ester being the most abundant, followed by alkanes and alkenes, accounting for 23.3%, 18.4%, and 17.5% of the total compounds, respectively.

**Fig. 2:**
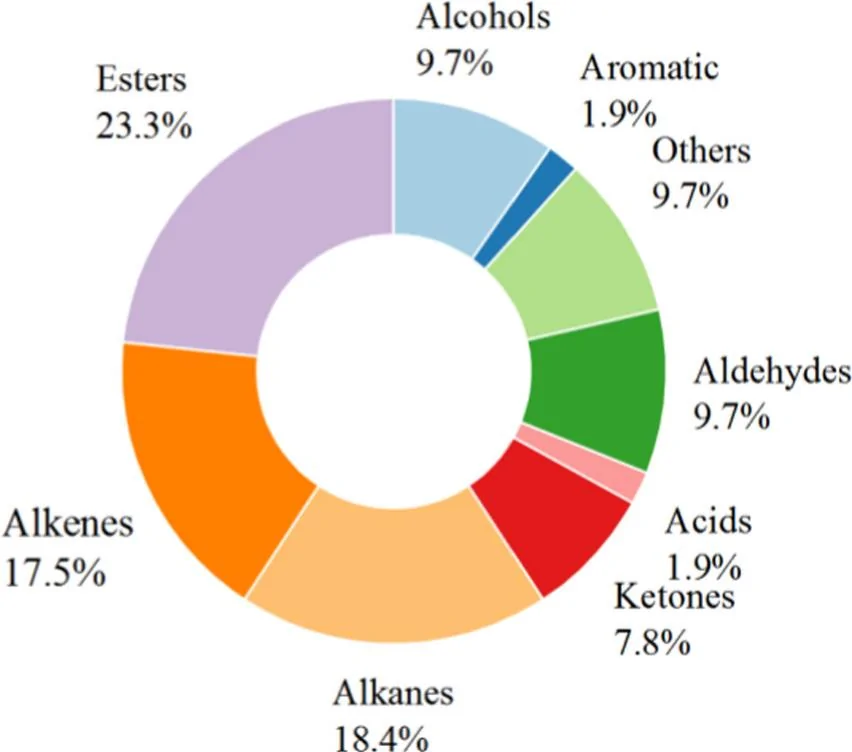
Analysis of shared volatile flavor compounds in Layan 12.

The number and concentration of volatile compound types varied significantly among different producing areas. As shown in Table 1, the number and percentage of compounds that accounted for the larger proportion in Layan 12 planted in four areas were different, with a higher number and percentage of alkanes in B2, aldehydes in B6, more alkanes and a higher percentage of alkenes in B7, and a higher number and percentage of esters in B8. The number and percentage of esters in B8 were higher than those in other areas. Esters exhibit fruity or distinctive aromatic notes, while aldehyde possess lower aroma threshold and stronger aroma-imparting capacity, with most presenting fruit, oil or grassy characteristics. Ketones typically impart mushroom, milky, or floral fragrances, whereas heterocyclic compounds are characterized by nutty or roasted aromas (Jia et al., 2025). For instance, methyl laurate and methyl salicylate, which have mushroom and mint scents respectively, are found in greater amounts in B8. Isovaleraldehyde, known for its floral scent, is more concentrated in B2, followed closely by B8. Additionally, dihydro-beta-ionone, which has an earthy aroma, is also more prevalent in B2. Lastly, 2-acetylpyrrole, recognized for its burnt scent, is found in higher concentrations in B2, with B8 following.

**Table 1:** Types and relative contents of volatile flavor compounds in Layan 12 from different producing areas.

Aroma component	B2		B6		B7		B8	
	Kinds	Contents (%)	Kinds	Contents (%)	Kinds	Contents (%)	Kinds	Contents (%)
Alcohols	2	0.99	2	9.16	3	1.97	5	10.8
Aromatic hydrocarbons	1	2.91	1	0.76	1	0.29	1	0.7
Others	4	8.95	5	24.25	7	7.39	5	16.68
Aldehydes	2	5.59	7	22.61	6	6.4	7	18.2
Acids	0	0	0	0	1	1.93	2	6.22
Ketones	5	8.28	1	2.33	4	0.56	2	8.12
Alkanes	9	67.67	6	14.73	13	11.15	9	10.7
Alkenes	6	4.45	5	8.83	9	60.07	8	8.06
Esters	3	1.17	7	17.34	17	10.26	19	20.51
Total	32	100	34	100	61	100	58	100

With 103 volatile flavor compounds as the dependent variable and different producing areas as the independent variables, the distinction between the four areas was basically achieved by OPLS-DA (Fig.3A). The fit index (R2x) for the independent variable was 0.987, the fit index (R2y) for the dependent variable was 0.997, and the model prediction index (Q2) was 0.995, with R2 and Q2 exceeding 0.5 to indicate an acceptable model fit. After 200 replacement tests，as shown in Fig.3, the intersection point of the Q2 regression line and the vertical axis was less than 0, indicating that there was no over-fitting of the model, and the model was verified to be valid, and it was considered that the results could be used for the identification and analysis of the volatile flavor compounds in different producing areas of Layan 12. The OAV can more accurately explain the influence of volatile components on the aroma compared to the content of volatile components, and the higher the OAV, the greater the contribution to the aroma (Acree et al., 1984), where OAV > 1 indicated that the volatile component had a certain impact on the food, and OAV > 10.0 indicated that the volatile component had a significant contribution to the overall aroma (Du et al., 2011). Variable importance projection (VIP) was used as a screening criterion to assess the contribution of metabolites to the classification of the samples (Feng et al., 2022).

**Fig. 3:**
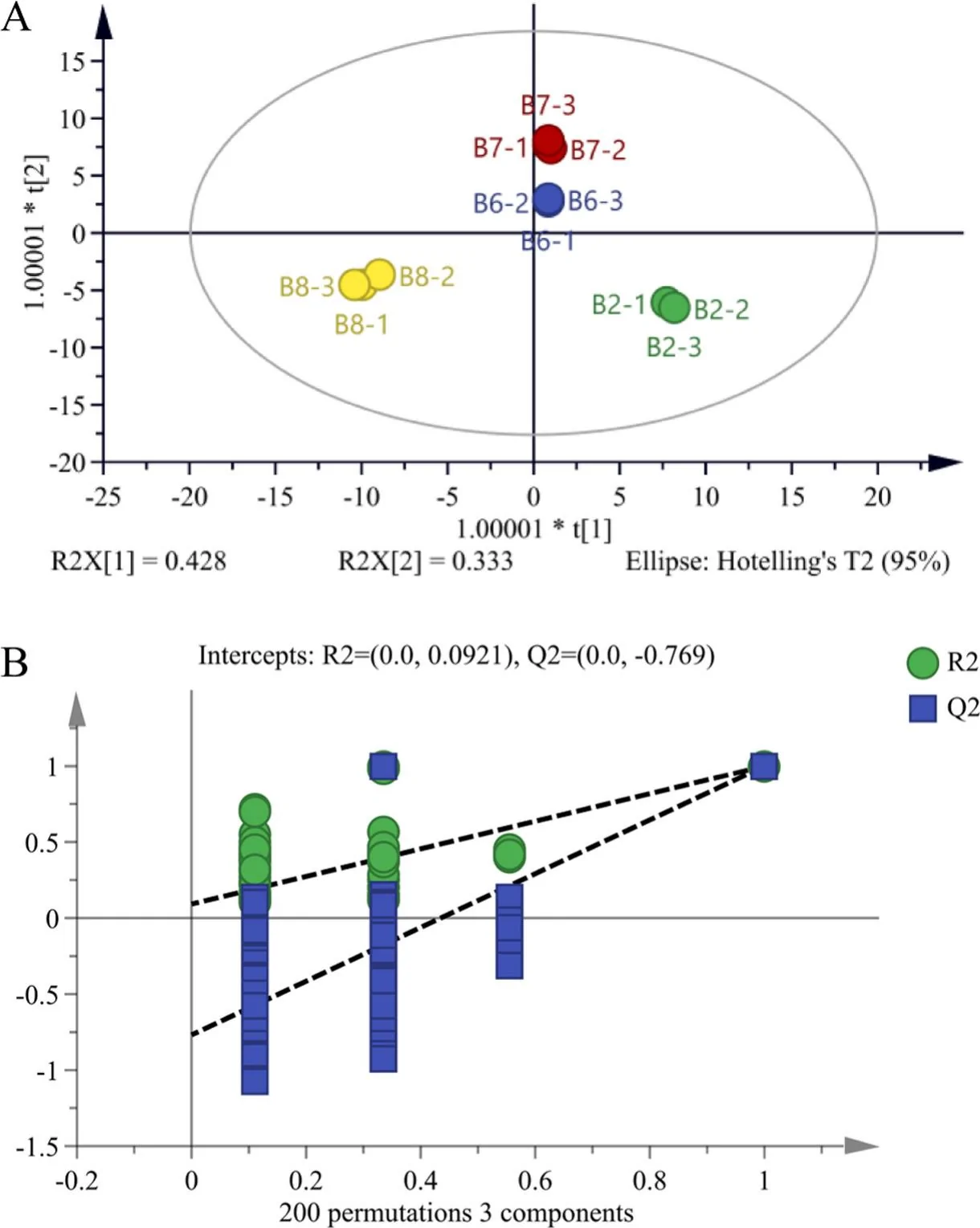
The analyses of volatile flavor compounds in Layan 12 from different producing areas. (A) Principal component analysis of volatile flavor compounds in Layan 12, (B) Results of 200 replacement tests.

Using VIP > 1 and OAV > 1 as screening criteria, 11 differential volatile flavor compounds contributing to the aroma of Layan 12 were identified across the four producing areas: d-limonene, nonanal, benzyl alcohol, hexanal, ylangene, longifolene, beta-Ionone, dodecane, styrene, beta-cyclocitral and dihydroactinidiolide. The composition and content of volatile flavor compounds varied significantly among the planting sites. Notably, B8 contained higher beta-cyclocitral and dihydroactinidiolide (herbal and coumarin-like odors, respectively) than other areas, and also exhibited a high level of nonanal with a greenish aroma (Wang et al., 2023; Yuan et al., 2025) (See Table 2).

**Table 2:** OAV values of volatile flavor compounds in different producing areas for Layan 12 (VIP > 1, OAV > 1).

Name	B2	B6	B7	B8	Green aroma	Fragrance type
d-Limonene	–	27.57 ± 3.29	–	–	Floral aroma, fruity aroma	Citrus
Nonanal	995.22 ± 59.04	2184.77 ± 274.41	157.73 ± 13.26	1275.27 ± 189.55	Pungent aroma	Aldehydic
Benzyl alcohol	–	1.92 ± 0.22	0.56 ± 0.07	–	–	Floral
Hexanal	–	1078.59 ± 89.26	–	700.49 ± 82.94	Sweet, woody aroma, rose aroma, pine needle aroma	Green
Ylangene	–	–	223.13 ± 24.2	–	Floral aroma, woody aroma, sweet fruity aroma, berry aroma	–
Longifolene	–	–	1.1 ± 0.16	–	Pungent aroma	Woody
beta-Ionone	9315.94 ± 1242.6	10,953.65 ± 1321.17	–	–	Balsamic aroma	Floral
Dodecane	3 ± 0.07	2.93 ± 0.31	–	–	Herbal, rose oxide, tobacco damascenone fruity aroma	–
Styrene	30.08 ± 3.48	–	4.84 ± 0.57	11.47 ± 1.39	Coumarin aroma	Balsamic
beta-Cyclocitral	–	209.78 ± 30.05	45.93 ± 4.81	218.39 ± 28.44	Green aroma	Tropical
Dihydroactinidiolide	–	663.25 ± 38.77	–	842.5 ± 101.57	Floral aroma, fruity aroma	–

### 3.3 Untargeted metabolomics analysis

#### 3.3.1 Methodological evaluation

To ensure method accuracy, a correlation coefficient diagram between the samples was constructed (Fig.4). The R^2^ values were ≥ 0.8, indicating good inter-sample correlation. The established pretreatment and detection method proved stable and the reliability, and was therefore suitable for untargeted metabolites screening and multivariate statistical analysis of chili samples. Based on the test results of chili samples from different producing areas, PCA based on the test results of chili samples from different producing areas was performed (Fig. 5.), which showed obvious clustering within groups and clear separation between group, with no overlap, indicating significant inter-groupdifferences. The Venn diagram indicated the number of shared and unique metabolites between groups, with a total of 3419 metabolites in the four areas (Among them, 521 compounds were annotated at MSI Level 2), 11, 6, and 17 metabolites shared by B2 and B8, B6 and B8, B7 and B8, respectively, and 8, 10, 34, and 11 metabolites unique to B2, B6, B7, and B8, respectively. The reason only MSI Level 2 and Level 3 assignments were reported is that untargeted metabolomics, as a semi-quantitative analytical approach, did not employ authentic standards. Consequently, the identified compounds could not meet the criteria for MSI Level 1 identification, which requires confirmation using reference standards.

**Fig. 4:**
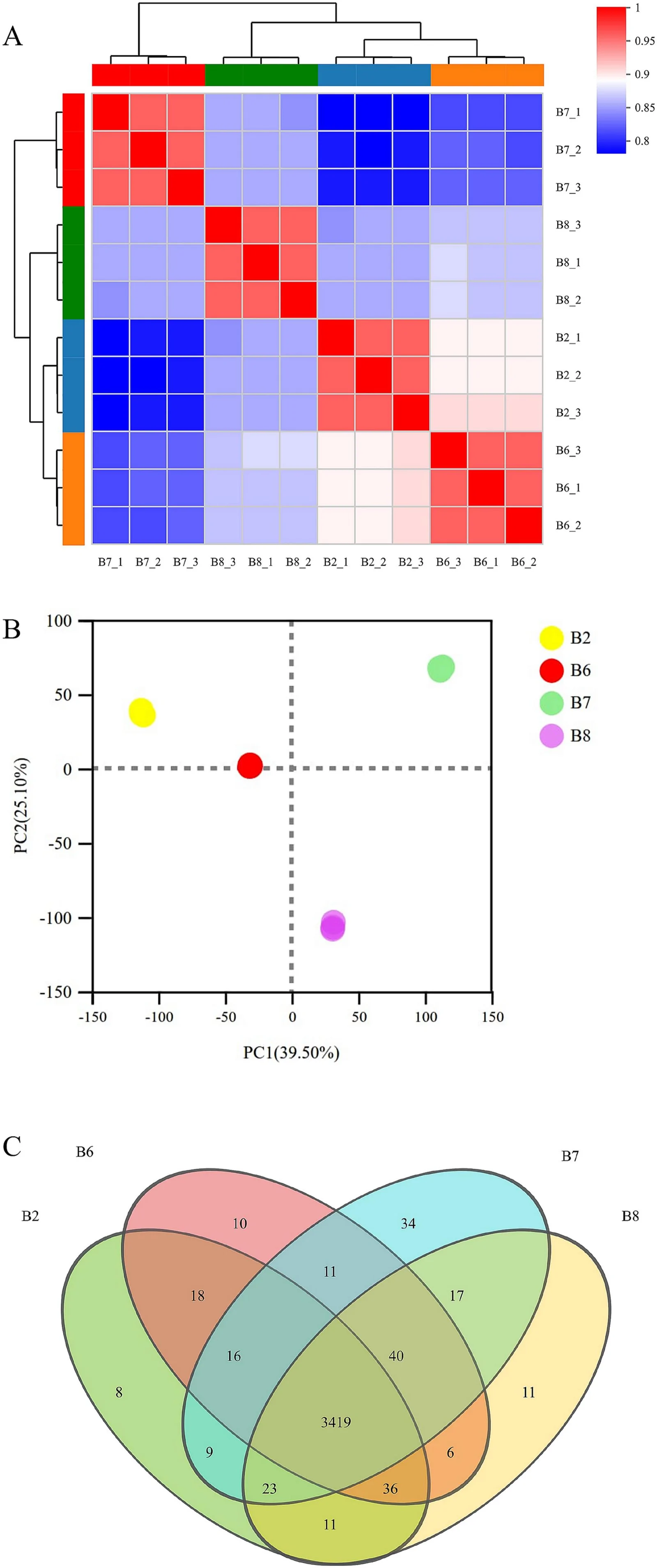
Untargeted metabolite analysis chart for Layan 12 grown in different producing areas. (A) Inter-sample correlation analysis, (B) Inter-sample principal component analysis, (C) Untargeted metabolite Venn diagram.

**Fig. 5:**
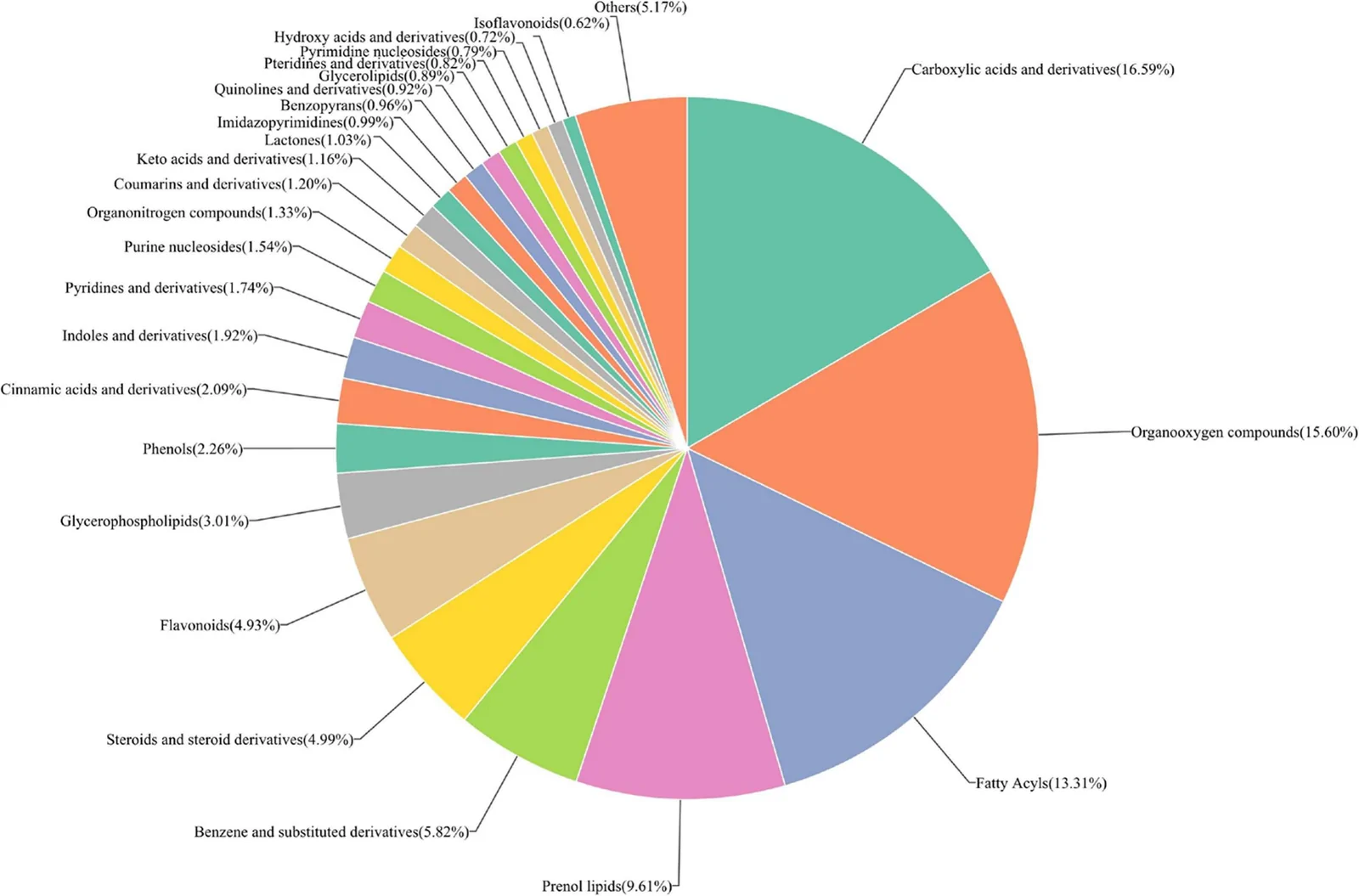
Types of metabolites annotated by Layan 12.

#### 3.3.2 Metabolite species analysis

The metabolite profiling of chili samples was carried out using UPLC-MS/MS. n positive ion mode, 8168 mass spectral peaks were detected and 1905 compounds were annotated; in negative ion mode,10,525 peaks were detected and 1746 compounds were annotated. In total, 3651 metabolites were annotated across the two ion modes. The annotated metabolites included carboxylic acids and derivatives (485 species), organooxygen compounds (456 species), fatty acyls (389 species), prenol lipids (281 species), benzene and substituted derivatives (170 species), steroids and steroid derivatives (146 species), as shown in Fig.5.

#### 3.3.3 Differential metabolites analysis

Differential metabolites were determined by VIP > 1; *P* < 0. 05. Table 3 shows the classification of the top 20 differential metabolite quantities in different producing areas of Layan 12, in which organooxygen compounds, carboxylic acids and derivatives, fatty acyls, prenol lipids, benzene and substituted derivatives, steroids and steroid derivatives, were more in the top of the list in terms of the number of differential metabolite quantities and their percentage.

**Table 3:** Differential metabolite classification analysis of Layan 12 from different producing areas.

Categories of differential metabolites	Kinds (Contents%)		
	B2–B8	B6–B8	B7–B8
Organooxygen compounds	135(12.99)	117(11.02)	137(11.77)
Carboxylic acids and derivatives	126(12.13)	132(12.43)	136(11.68)
Fatty Acyls	120(11.55)	136(12.81)	131(11.25)
Prenol lipids	88(8.47)	85(8)	95(8.16)
Benzene and substituted derivatives	52(5)	45(4.24)	59(5.07)
Steroids and steroid derivatives	52(5)	54(5.08)	47(4.04)
Flavonoids	43(4.14)	48(4.52)	44(3.78)
Glycerophospholipids	27(2.6)	21(1.98)	27(2.32)
Cinnamic acids and derivatives	21(2.02)	26(2.45)	21(1.8)
Phenols	20(1.92)	19(1.79)	23(1.98)
Indoles and derivatives	19(1.83)	18(1.69)	19(1.63)
Quinolines and derivatives	13(1.25)	10(0.94)	8(0.69)
Pyridines and derivatives	12(1.15)	14(1.32)	12(1.03)
Purine nucleosides	12(1.15)	9(0.85)	8(0.69)
Coumarins and derivatives	10(0.96)	9(0.85)	17(1.46)
Benzopyrans	9(0.87)	13(1.22)	10(0.86)
Organonitrogen compounds	9(0.87)	10(0.94)	14(1.2)
Macrolides and analogues	8(0.77)	9(0.85)	9(0.77)
Lactones	8(0.77)	9(0.85)	10(0.86)
Others	255(24.54)	278(26.18)	337(28.95)
Total	1039(100)	1062(100)	1164(100)

The volcano diagram of the differential metabolites in different producing areas of Layan 12 are shown in Fig. 6. From the figure, the number of significant different metabolites of B2-B8, B6-B8 and B7-B8 were 1039, 1062 and 1164, respectively. More than 50% of the metabolites were significantly down-regulated between B2-B8, B6-B8 and B7-B8 samples, with the down-regulated metabolites of B7-B8 accounting for 62.37%, the down-regulated metabolites mainly included fatty acyls, organooxygen compounds, carboxylic acids and derivatives, prenol lipids, steroids and steroid derivatives, isoprenol lipids and steroids and steroid derivatives, suggesting that these groups of metabolites have a high content in B8.

**Fig. 6:**
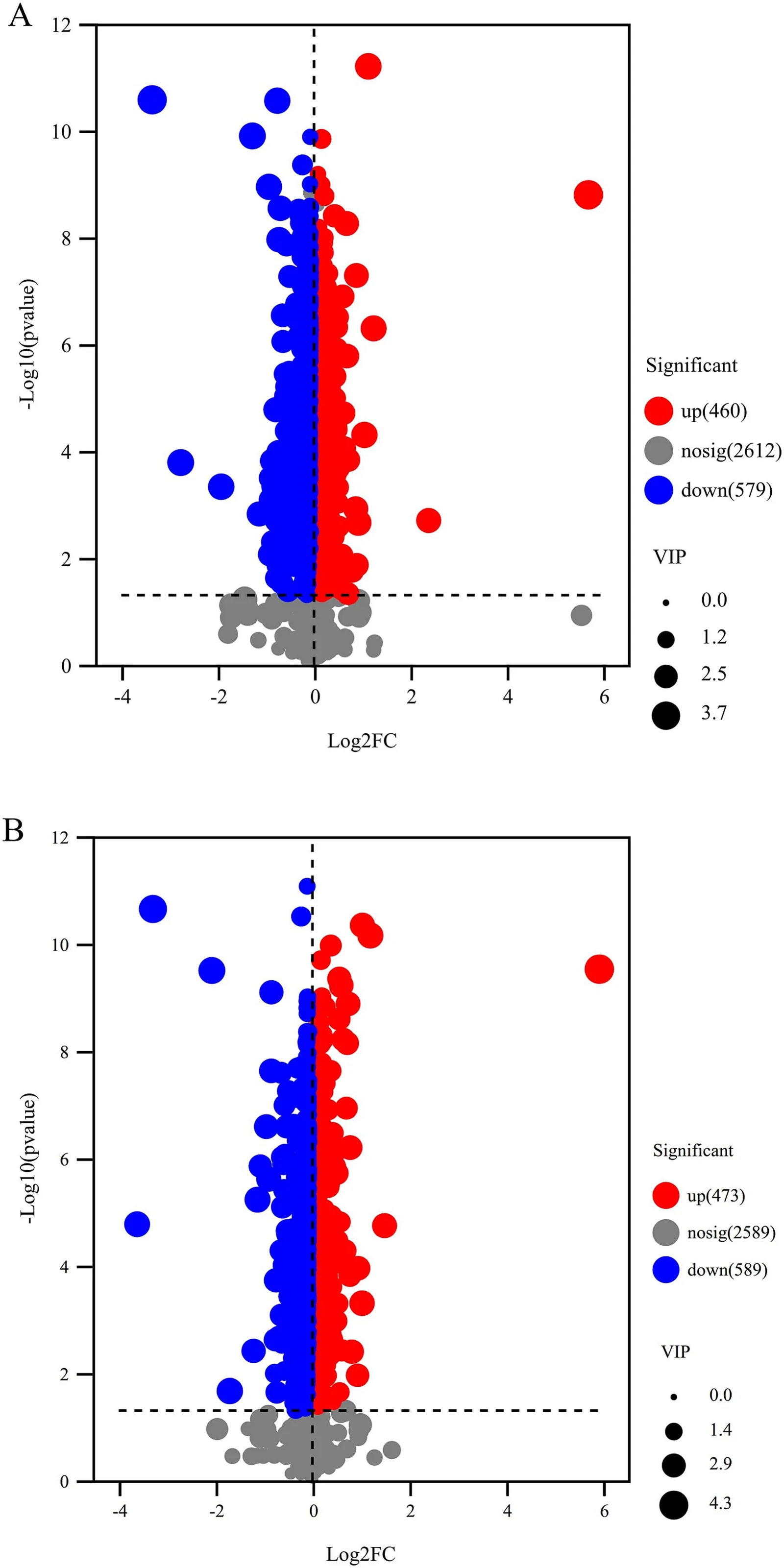

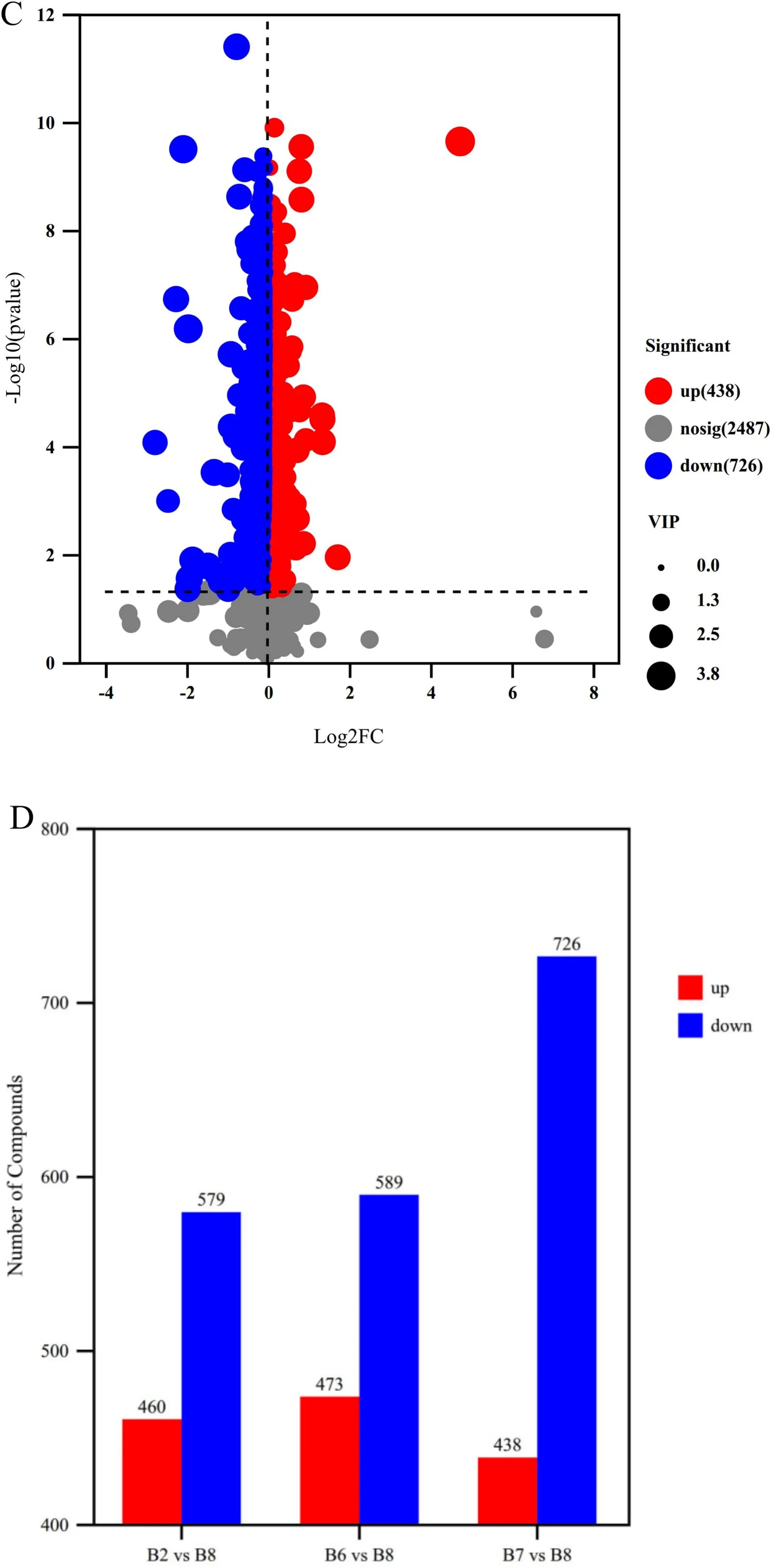
Differential analysis of metabolites for Layan 12 grown in different producing areas. (A) Volcano of B2 vs B8, (B) Volcano of B6 vs B8, (C) Volcano of B7 vs B8, (D) Significant difference.

#### 3.3.4 Differential metabolite pathway enrichment

Pathway enrichment analysis was performed using the KEGG database for the B2-B8, B6-B8, and B7-B8 comparisons, focusing on the top 20 metabolites. As shown in Fig. 7, the significant differential metabolites of Layan 12 from different producing areas were mainly distributed in six metabolic pathways, namely, linoleic acid metabolism, flavone and flavonol biosynthesis, alpha-Linolenic acid metabolism, flavonoid biosynthesis, Arachidonic acid metabolism, Glycerophospholipid metabolism.

**Fig. 7:**
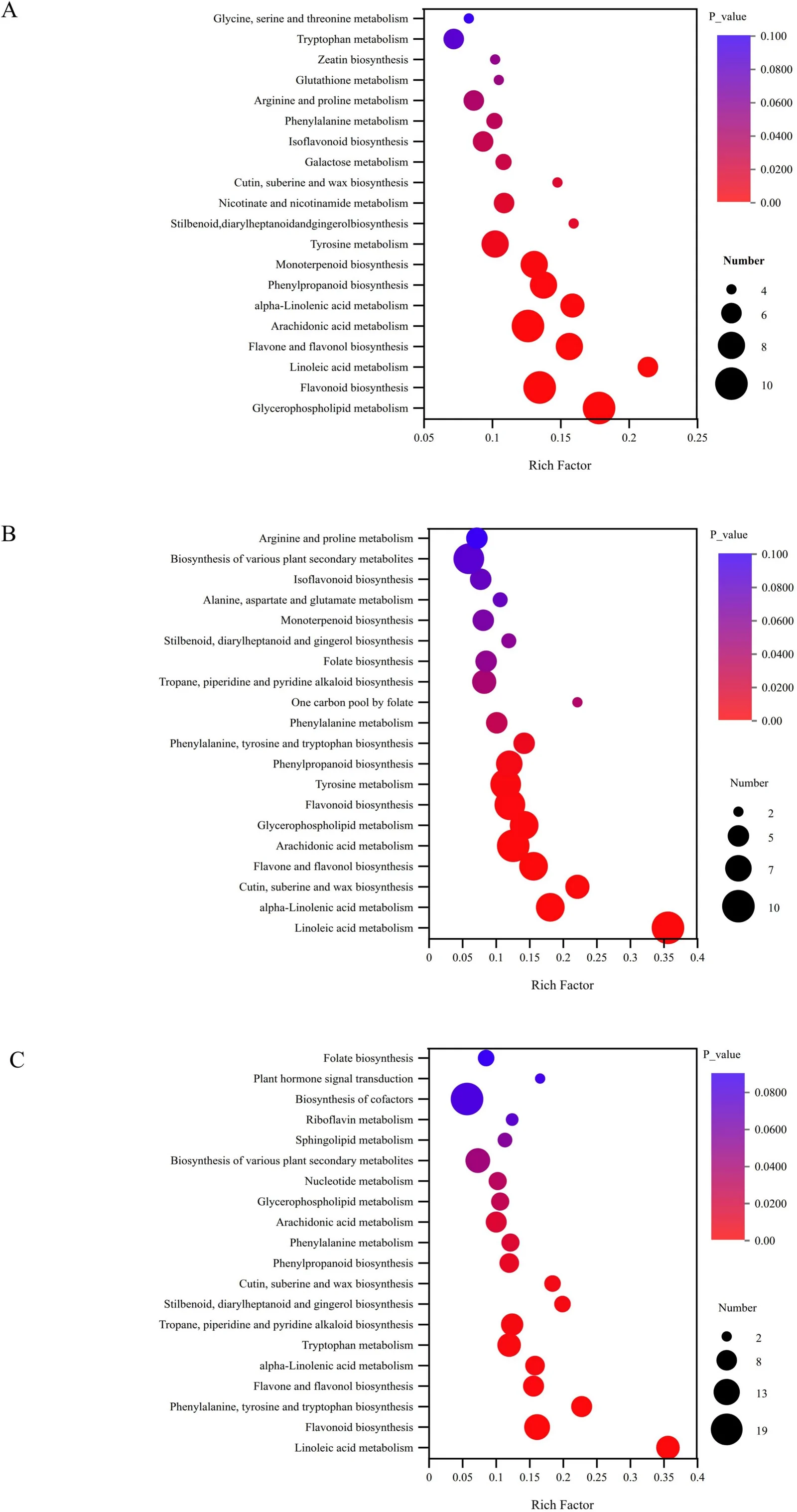
KEGG Enrichment Analysis for Layan 12 grown in different producing areas. (A) B2 vs B8, (B) B6 vs B8, (C) B7 vs B8.

#### 3.3.5 Analysis of key characteristic components

Key characteristic components were annotated based on differential metabolites with annotation confidence levels (MSI levels) 2, VIP > 2, *P* < 0. 01, FC > 1.5 or < 0.67. As shown in Table 4, Layan 12 from different producing areas contained 5 key characteristic components: 2 between B2 and B8, and 3 each between B6 and B8 and between B7 and B8.

**Table 4:** Key characteristic components of Layan 12 from different producing areas based on untargeted metabolomics.

No.	Grouping	Key characteristic components	VIP	FC	Relative concentration	*M*/*Z*	Retention time/min	Addition ion	Chemical formula
1	B2–B8	Thymyl acetate	2.73	0.54	↓	193.12	4.10	[M + H-2H_2_O, M + H]^+^	C_12_H_16_O_2_
2	B2–B8	Tryptophol	2.21	0.64	↓	203.12	5.61	[M + ACN + H]^+^	C_10_H_11_NO
3	B6–B8	Thymyl acetate	2.59	0.65	↓	193.12	4.10	[M + H-2H_2_O, M + H]^+^	C_12_H_16_O_2_
4	B6–B8	Ala Lys Leu Asp	2.58	1.71		446.26	4.03	[M + H]^+^	C_19_H_35_N_5_O_7_
5	B6–B8	Tryptophol	2.36	0.65	↓	203.12	5.61	[M + ACN + H]^+^	C_10_H_11_NO
6	B7–B8	Quercetin	2.74	1.72		303.05	5.62	[M + H]^+^	C_15_H_10_O_7_
7	B7–B8	Ala Lys Leu Asp	2.68	1.85		446.26	4.03	[M + H]^+^	C_19_H_35_N_5_O_7_
8	B7–B8	Isopentenyl-Adenine-9-Glucoside	2.39	1.53		366.18	5.54	[M + H]^+^	C_16_H_23_N_5_O_5_

The cluster heatmap of key characteristic components of Layan 12 grown in different producing areas (Fig. 8) shows that geographical origin exerts a significant influence on the accumulation of specific metabolites. Samples from different producing areas (B2, B6, B7, B8) formed distinct clusters, indicating clear differences in their metabolic profiles.

**Fig. 8:**
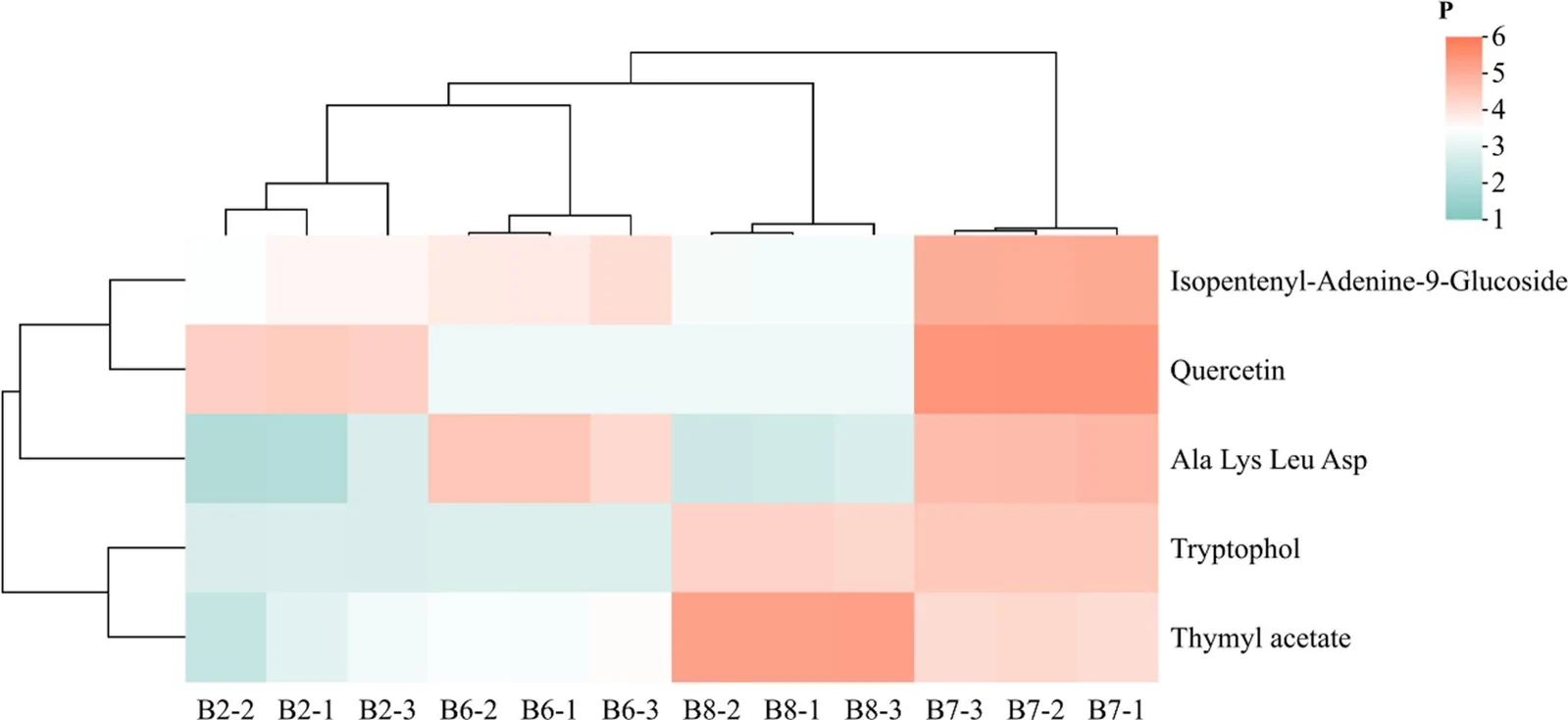
Cluster heat map of key characteristic components of Layan 12 grown in different producing areas.

Among them, isopentenyl-adenine-9-glucoside, a cytokinin derivative involved in plant growth and stress responses, was present at higher levels (dark orange region) in samples from the B7 area, significantly exceeding those in other regions. This may suggest that the unique environmental conditions of B7—such as temperature, light, or water availability—promoted the accumulation of this compound by enhancing cytokinin signaling pathways in response to local abiotic stresses. A similar trend was observed for quercetin, a common antioxidant flavonoid, which showed higher abundance (yellow to orange region) in B7 samples, while its levels were lower (blue to green region) in B2 and B6 samples. This result is consistent with previous findings that flavonoid accumulation in plants is significantly influenced by environmental factors such as UV radiation and temperature fluctuations.

In contrast, the metabolic profile of the B8 area was markedly distinct from those of the other three regions. In B8 samples, the contents of the amino acids alanine (Ala), lysine (Lys), leucine (Leu), and aspartic acid (Asp) were all lower than those in the B2, B6, and B7 areas. This differential accumulation of free amino acids may reflect specific variations in nitrogen metabolism or protein turnover rates in the B8 area, which could be regulated by local soil nutrient conditions or climatic factors. Notably, tryptophol (a plant auxin-related metabolite) and thymyl acetate (a volatile ester compound) also displayed distribution patterns in B8 samples that differed from those in other regions, further supporting the notion that environmental factors drive metabolic differentiation in chili fruits. Moreover, compared with the B7 area, which was enriched in isopentenyl-adenine-9-glucoside and quercetin, the B8 area exhibited lower overall levels of most key characteristic components, forming a distinct region-specific metabolic profile. Overall, the metabolic profiles of B2 and B6 were relatively similar, while B7 and B8 each presented unique metabolic characteristics. These results indicate that environmental conditions across different producing areas exert a significant differentiation effects on the accumulation of primary and secondary metabolites in Layan 12, with B8 exhibiting a particularly pronounced metabolic signature.

### 3.4 Analysis of meteorological factors

#### 3.4.1 Comparison of meteorological factors

As shows in Table 5, climatic conditions varied across the four planting locations during the growth phase. Temperatures ranked from highest to lowest in the order B2 > B7 > B6 > B8. The B2 site has a temperate continental arid climate, characterized by minimal cloud cover, dry air, and direct sunlight, leading to notably daytime maximum temperatures. In contrast, B7 features a warm, humid monsoon climate, benefiting from its lower latitude and higher solar angle, which results in substantial solar exposure and elevated temperatures. B6, with a temperate continental monsoon climate, has a higher latitude and less intense solar radiation, resulting in lower maximum temperatures compared to B2 and B7, despite its elevation being similar to that of B2. Although B8 has a humid subtropical monsoon climate, its significant elevation, coupled with ample cloud cover and rainfall, diminishes solar radiation, thereby reducing daytime heating and leading to lower maximum temperatures. The variation in daily temperatures is ranked as B6, B2, B7, and B8. In B6, the combination of dry, clear weather and moderate elevation facilitates rapid warming during the day and significant cooling at night, creating a wide temperature range. While B2 records higher daytime temperatures, its position on the southern fringe of the Junggar Basin—a relatively enclosed area—promotes the development of an inversion layer at night, resulting in higher minimum temperatures; thus, its temperature range is slightly narrower than that of B6. The cumulative effective temperature ≥ 10 °C is ranked from highest to lowest as B7, B2, B8, and B6. B7, due to its low latitude and elevation, experiences an early onset of spring warmth and a delayed autumn cool-down, leading to an extended duration of temperatures at or above 10 °C. Despite B8's low latitude, its high elevation results in cooler temperatures, restricting the accumulation of effective temperature. The average relative humidity and the share of diffuse radiation are ranked as B8, B7, B6, and B2. B8 is affected by warm, moist air currents, resulting in significant precipitation, high humidity, and dense cloud cover, which contribute to a high proportion of diffuse radiation. Conversely, B2, with its arid climate, has the lowest humidity and the least amount of diffuse radiation. The total radiation levels are ranked as B2, B6, B7, and B8. B2, being arid with minimal cloud cover, receives the highest total radiation; B6 enjoys more sunny days, resulting in the second-highest total radiation; while B7 and B8, influenced by monsoon rains, experience increased cloud cover and consequently lower total radiation.

**Table 5:** Meteorological data of different planting areas during the field growth period.

Meteorological factor	B2	B6	B7	B8
The highest temperature/°C	40.95	38.54	40.02	32.92
Diurnal temperature range/°C	11.01	12.52	8.99	7.16
Effective accumulated temperature ≥ 10 °C/°C	2034.20	1788.30	2452.95	1926.17
Relative humidity/%	38.53	50.00	69.15	78.72
Global radiation/MJ/m^2^	3351.13	3119.19	2838.02	2387.66
Ratio of diffuse Radiation/%	71.61	75.45	79.63	86.47

#### 3.4.2 Correlation between quality indicators and meteorological factors

Based on the above analysis, 19 indicators were selected as quality indicators: 8 physicochemical indicators, 6 flavor indicators (from differential volatile flavor compounds with OAV > 100), and 5 metabolic indicators (from differential metabolites with VIP > 2. A correlation analysis was conducted between these indicators and 6 meteorological factors, as shown in Fig. 9.

**Fig. 9:**
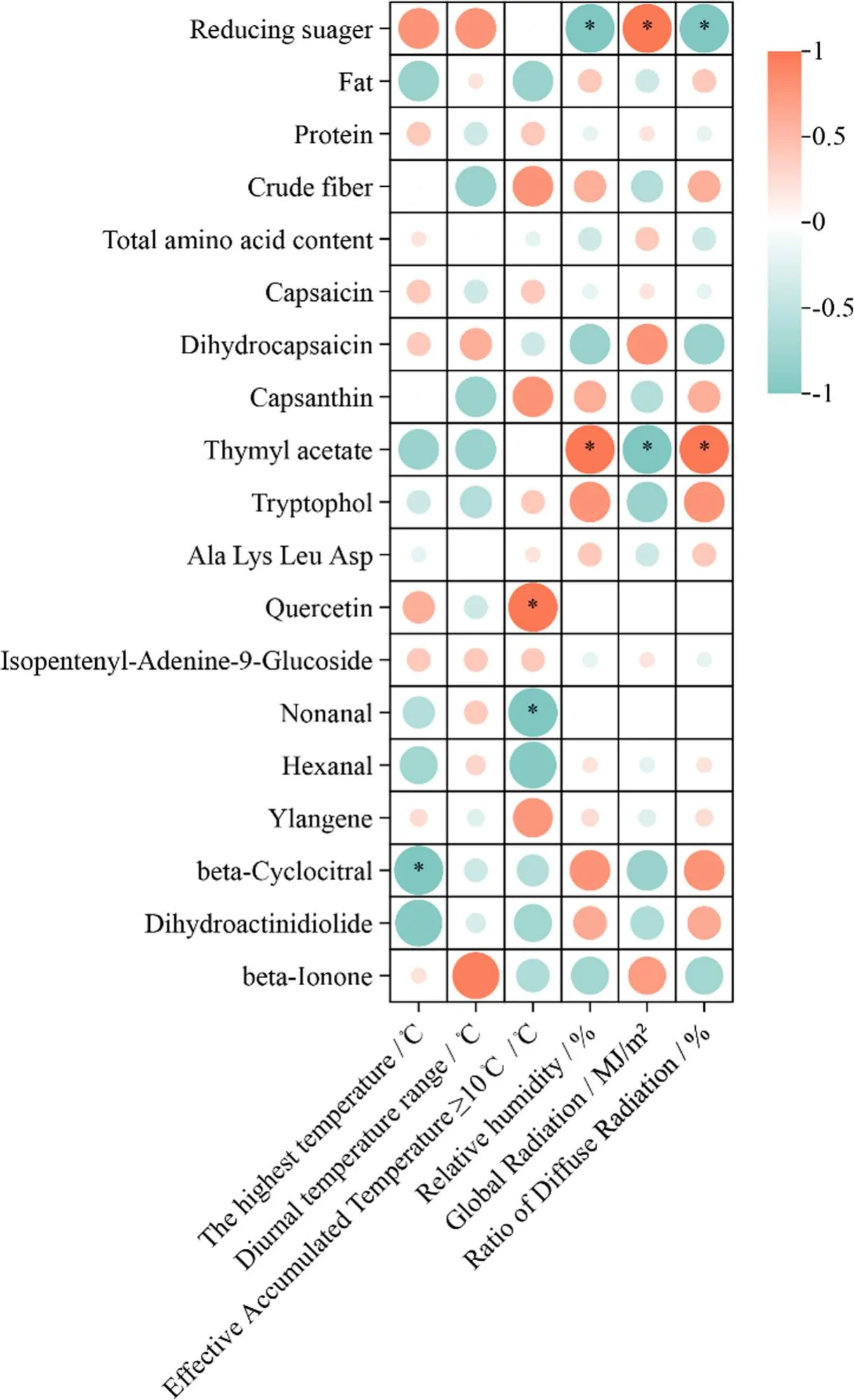
Correlation analysis of quality indexes of Layan 12 from different planting regions with meteorological factors based on Spearman algorithm (* indicated significant correlation (*P* < 0.05)).

Spearman correlation analysis revealed varying degrees of association between the physicochemical indices, metabolites, and volatile flavor compounds of Layan 12 and multiple meteorological factors, indicating that environmental conditions exert multi-dimensional regulatory effect on chili quality formation.

Associations between physicochemical indices and meteorological factors: Among the physicochemical indices, reducing sugar was significant positively correlated with global radiation and negatively correlated with relative humidity and the diffuse radiation ratio (*P* < 0.05). This suggests that ample global radiation may be associated with the accumulation of photosynthetic products, whereas high humidity and a high diffuse radiation ratio (often accompanying cloudy and rainy weather) may be related to a reduction in sugar accumulation. Crude fat was negatively correlated with maximum temperature and ≥ 10 °C effective accumulated temperature, indicating that excessive thermal conditions may not favor fat accumulation, a trend differing from the positive associations observed for protein and capsaicin. Among the capsaicinoids, dihydrocapsaicin was positively correlated with global radiation, suggesting that stronger radiation conditions may be related to its synthesis, possibly by providing abundant photosynthetic carbon sources. Capsanthin was primarily associated with temperature factors, showing a negative correlation with diurnal temperature range and a positive correlation with ≥10 °C effective accumulated temperature, implying that capsanthin synthesis may respond differently to total heat accumulation and temperature fluctuations.

Associations between key metabolites and meteorological factors: The accumulation of metabolites also showed pronounced environmental dependence. Isopentenyl-adenine-9-glucoside (a cytokinin derivative) was positively correlated with relative humidity and negatively correlated with diurnal temperature range, indicating that humid conditions may be associated with the accumulation of this compound. Quercetin showed a significant positive correlation with ≥10 °C effective accumulated temperature, suggesting that adequate thermal conditions are conducive to the synthesis of this antioxidant flavonoid. Among the amino acids, alanine, leucine, lysine, and aspartic acid were present at lower levels in the B8 region and were negatively correlated with global radiation. This may be related to the influence of light conditions across different regions on nitrogen metabolism or protein turnover rates. Notably, thymyl acetate (a volatile ester) was significantly negatively correlated with relative humidity but positively correlated with global radiation, indicating that dry, high-radiation environments may favor its accumulation—a trend similar to that observed for reducing sugar.

Associations between flavor compounds and meteorological factors: Flavor compounds exhibited divergent responses to meteorological factors. Nonanal and hexanal, both typical products of lipid oxidation, were negatively correlated with maximum temperature and effective accumulated temperature, suggesting that high thermal conditions may accelerate the further degradation of these aldehydes. Ylangene, a sesquiterpene, was positively correlated with effective accumulated temperature, indicating that total heat accumulation is a key factor influencing its accumulation. β-Cyclocitral and dihydroactinidiolide, both carotenoid degradation products, were negatively correlated with maximum temperature but positively correlated with relative humidity and the ratio of diffuse radiation. This implies that mild, humid environments with predominantly diffuse light may be associated with their accumulation. In contrast, β-ionone, also a carotenoid degradation product, was positively correlated with diurnal temperature range and global radiation, suggesting that its formation pathway may differ from that of the former two compounds, potentially requiring stronger light and greater temperature fluctuations to trigger specific oxidative cleavage reactions.

Through Spearman correlation analysis, this study systematically revealed the co-variation relationships between the physicochemical indices, key metabolites, and flavor compounds of Layan 12 and multiple meteorological factors across different planting regions. The results indicate that global radiation, relative humidity, temperature, and diurnal temperature range are important environmental factors associated with the formation of chili fruit quality. Among these, reducing sugar, thymyl acetate, and certain flavor compounds were more sensitive to radiation and humidity conditions, while protein, capsaicinoids, and some carotenoid degradation products were more closely associated with temperature conditions. The accumulation of different metabolites exhibited differentiated environmental response patterns, which also helps to explain why the B2 and B6 regions, as well as the B7 and B8 regions, formed distinct clustering characteristics in their metabolic profiles.

### 3.5 Establishment of a quantitative quality evaluation index system for production areas

#### 3.5.1 Selection of evaluation indicators

Based on the above analysis, eight chemical indexes (reducing sugar, fat, protein, crude fiber, total amino acid, capsaicin, dihydrocapsaicin and capsanthin) were selected as quality indicators. Six volatile flavor compounds with OAV>100 (nonanal, hexanal, ylangene, beta-cyclocitral, dihydroactinidiolide, beta-Ionone) were also included, along with five metabolite indicators (Thymyl acetate, Tryptophol, Ala Lys Leu Asp, Quercetin, Isopentenyl-Adenine-9-Glucoside). Principal component analysis was performed on a total of 19 indicators.

#### 3.5.2 Comprehensive scoring model construction

The components with the eigenvalues greater than 1 were selected as principal components. As shown in Table 6, the eigenvalues of three principal components were greater than 1, and the cumulative variance contribution rate was 100% (generally it was a satisfactory result that the cumulative variance contribution rate was greater than 70%), indicating that these three factors could represent all the information of 19 indicators of four samples, so these three principal components were selected for comprehensive evaluation. Among them, the contribution rate of variance of the first principal component was 41.569%, mainly reflecting total amino acid content, capsaicin, dihydrocapsaicin, capsanthin, Ylangene, beta-Ionone, Tryptophol and Isopentenyl-Adenine-9-Glucoside. The contribution rate of variance of the second principal component was 38.185%, which mainly reflected the information of fat, crude fiber, Nonanal, Hexanal, beta-Cyclocitral, Dihydroactinidiolide and Quercetin. The contribution rate of variance of the third principal component was 20.246%, which mainly reflected the information of reducing suager, protein, Thymyl acetate and Ala Lys Leu As. The principal component loads of each index are shown in Table 7.

**Table 6:** Eigenvalues and variance contributions of principal components.

Principal component	Eigenvalue	Variance contribution rate/%	Cumulative contribution rate/%
1	7.898	41.569	41.569
2	7.255	38.185	79.754
3	3.847	20.246	100

**Table 7:** Load matrix of principal components.

Indicators	Component 1		Component 2		Component 3	
	Load (U_1i_)	Factor score coefficient	Load (U_2i_)	Factor score coefficient	Load (U_3i_)	Factor score coefficient
A_1_ (Reducing suager)	−0.167	−0.470	−0.154	−0.414	−0.398	−0.780
A_2_ (Fat)	0.185	0.519	0.316	0.850	−0.048	−0.095
A_3_ (Protein)	−0.215	−0.605	−0.189	−0.508	0.313	0.614
A_4_ (Crude fiber)	0.113	0.318	−0.259	−0.698	0.327	0.641
A_5_ (Total amino acid content)	−0.340	−0.956	−0.063	−0.171	0.122	0.239
A_6_ (Capsaicin)	−0.256	−0.720	−0.236	−0.637	0.141	0.276
A_7_ (Dihydrocapsaicin)	−0.295	−0.829	−0.208	−0.559	0.017	0.034
A_8_ (Capsanthin)	0.297	0.835	−0.186	−0.500	0.117	0.229
A_9_ (Nonanal)	−0.209	−0.586	0.294	0.791	−0.091	−0.178
A_10_ (Hexanal)	−0.061	−0.172	0.365	0.982	−0.039	−0.076
A_11_ (Ylangene)	0.304	0.855	−0.161	−0.435	−0.145	−0.284
A_12_ (beta-Cyclocitral)	0.021	0.059	0.363	0.978	0.102	0.201
A_13_ (Dihydroactinidiolide)	−0.026	−0.073	0.345	0.929	0.186	0.364
A_14_ (beta-Ionone)	−0.287	−0.806	0.042	0.114	−0.296	−0.581
A_15_ (Thymyl acetate)	0.220	0.618	0.148	0.398	0.346	0.678
A_16_ (Tryptophol)	0.315	0.886	−0.019	−0.052	0.236	0.462
A_17_ (Ala Lys Leu Asp)	0.233	0.654	0.087	0.233	−0.367	−0.720
A_18_ (Quercetin)	0.172	0.483	−0.310	−0.834	−0.136	−0.267
A_19_ (Isopentenyl-Adenine-9-Glucoside)	0.260	0.732	−0.117	−0.315	−0.308	−0.604

After the standardization of each index, the three principal components were scored (Yi), i.e., the loadings (Ui) were multiplied by the standard values (Ai) of the 19 variables (Table 8), with higher scores indicating a greater contribution from the principal component.

**Table 8:** Calculating principal component scores.

Principal component	Principal component score calculation formula
1	Y1 = U_1i_A_1_ + U_1i_ A_2_+ U_1i_A_3_ + U_1i_ A_4_+ U_1i_A_5_ + U_1i_ A_6_+ U_1i_A_7_ + U_1i_ A_8_+ U_1i_ A_9_+ U_1i_A_10_ + U_1i_ A_11_ + U_1i_ A_12_+ U_1i_A_13_ + U_1i_ A_14_+ U_1i_A_15_ + U_1i_ A_16_+ U_1i_A_17_ + U_1i_ A_18_+ U_1i_ A_19_
2	Y2 = U_2i_A_1_ + U_2i_ A_2_+ U_2i_A_3_ + U_2i_ A_4_+ U_2i_A_5_ + U_2i_ A_6_+ U_2i_A_7_ + U_2i_ A_8_+ U_2i_ A_9_+ U_2i_A_10_ + U_2i_ A_11_ + U_2i_ A_12_+ U_2i_A_13_ + U_2i_ A_14_+ U_2i_A_25_ + U_2i_ A_16_+ U_2i_A_17_ + U_2i_ A_18_+ U_2i_ A_19_
3	Y3 = U_3i_A_1_ + U_3i_ A_2_+ U_3i_A_3_ + U_3i_ A_4_+ U_3i_A_5_ + U_3i_ A_6_+ U_3i_A_7_ + U_3i_ A_8_+ U_3i_ A_9_+ U_3i_A_10_ + U_3i_ A_11_ + U_3i_ A_12_+ U_3i_A_13_ + U_3i_ A_14_+ U_3i_A_15_ + U_3i_ A_16_+ U_3i_A_17_ + U_3i_ A_18_+ U_3i_ A_19_

#### 3.5.3 Ranking and validation of production areas

The eigenvectors were standardised, and then the inner product was made with the corresponding three principal component scores to obtain the evaluation function Y of the comprehensive quality, and the expression of Y is Y = 0.4157 × Y1 + 0.3819 × Y2 + 0.2025 × Y3. According to the comprehensive scores obtained from the formulae and the ordering (Table 9), the comprehensive scores of Layan12 in different producing areas were B8> B7> B6> B2 from high to low.

**Table 9:** Principal component score for Layan 12 from different producing areas.

Producing areas	Y1	Y2	Y3	Y	Scoring Rankings
B8	0.509	1.678	2.653	1.389	1
B7	3.603	−1.758	−0.835	0.658	2
B6	−1.053	2.857	−1.947	0.259	3
B2	−3.058	−2.777	0.128	−2.306	4

## 4 Conclusion

This study conducted a comprehensive analysis of Layan 12 grown in four different producing areas (Xinjiang, Inner Mongolia, Henan and Guizhou). The analysis assessed chemical parameters, volatile flavor compounds, untargeted metabolites, and meteorological factors to understand how growing location influences this particular chili variety. Findings revealed significant quality variations among the regions, with Guizhou's Layan 12 outperforming those from Henan, Xinjiang and Inner Mongolia. The study identified 103 volatile flavor compounds across the four areas, including 11 that were notably different, as well as 3419 metabolites comprising 16 categories and 5 key characteristic components. Metabolomic profiling combined with multivariate analysis successfully distinguished chili samples from different regions, providing a robust basis for understanding the environmental regulation of chili fruit quality. Moreover, this study presents a quantifiable quality evaluation index system for production areas, which integrates 19 indicators spanning physicochemical properties, volatile flavor compounds, and key metabolites into a principal component-based scoring model. This system enables objective ranking of production areas and offers a reproducible framework applicable to other chili varieties and regions. These results not only confirm the existence of region-specific metabolic signatures but also offer a theoretical foundation for selecting optimal planting areas to enhance the functional and sensory quality of chili.

However, this research primarily focused on a single variety (Layan 12) and did not account for the notable variations in adaptability among different chili varieties in response to environmental factors such as altitude, latitude, longitude, and climatic conditions. The limited variety selection represents a constraint of this study. Additionally, it should be noted that oven-drying at 60 °C may partially affect volatile compounds and thermolabile metabolites; therefore, the present results primarily reflect the quality of dried chili products. Future studies comparing fresh and oven-dried samples will help validate the robustness of the proposed quality evaluation system. Consequently, future studies should emphasize the essential indicators proposed in this paper, targeted validation of these key markers using authentic standards, and multi-omics integration to further elucidate the underlying regulatory mechanisms. Future studies should expand the sample size of chili varieties, integrate metabolomics with other analytical technologies, and conduct comprehensive investigations in the context of specific chili application scenarios. Such efforts will promote the optimization of chili and its derivatives across production areas.

## CRediT authorship contribution statement

**Wenxin Li:** Writing – review & editing, Writing – original draft, Visualization, Methodology, Data curation. **Yong Yin:** Writing – review & editing, Investigation, Formal analysis, Data curation. **Peng Zhou:** Data curation. **Jia Yan:** Methodology. **Yongping Wang:** Resources. **Xiaojing Sun:** Writing – review & editing. **Ju Chen:** Writing – review & editing. **Min Lu:** Investigation. **Xueya Wang:** Writing – review & editing, Validation, Resources, Conceptualization. **Jianwen He:** Writing – review & editing, Supervision, Resources, Project administration.

## Declaration of competing interest

The authors declare that they have no known competing financial interests or personal relationships that could have appeared to influence the work reported in this paper.

## Data Availability

Data will be made available on request.

## Appendix A Supplementary data

GC-MS Data

LC-MS PeakTable

LC-MS TIC BPC QC
