# Supplementary material for: Quantitative evaluation of quality indicators and flavor characteristics of the same variety of chili from different producing areas of China: An integrated multivariate analysis
Source: Food Chem X. 2026 Oct 7;39:104589. doi: 10.1016/j.fochx.2026.104589 (PMC13677961; doi:10.1016/j.fochx.2026.104589)
Supplement: Supplementary file 1 [file mmc1.docx]

**1 Total ion chromatograms (TIC) of samples from each production area obtained by GC-MS**

**1.1 B2-1**

**1.2 B6-1**

**1.3 B7-1**

**1.4 B8-1**

**2 Mass spectra of key compounds**

**1.1 Nonanal**

**1.2 Hexanal**

**1.3 Ylangene**

**1.4 beta-Cyclocitral**

**1.5 Dihydroactinidiolide**

**1.6 beta-Ionone**

| Compound Name | Component RT | Library RI | Experimental RI | Peak area | | | | | | | | | | | |
| --- | --- | --- | --- | --- | --- | --- | --- | --- | --- | --- | --- | --- | --- | --- | --- |
|  |  |  |  | B2-1 | B2-2 | B2-3 | B6-1 | B6-2 | B6-3 | B7-1 | B7-2 | B7-3 | B8-1 | B8-2 | B8-3 |
| Trichloromethane | 4.09085 | 573 | 646.06858 | 2525031439 | 2860240573 | 2554612720 | - | - | - | - | - | - | - | - | - |
| Butanal, 3-methyl- | 4.84328 | 643 | 671.7838 | 232508678.1 | 265398172.8 | 218584651.2 | - | - | - | 654887.246 | 668414.3916 | 663636.3882 | 13599524.01 | 18530577.7 | 14448282.65 |
| Butanal, 2-methyl- | 5.02484 | 643 | 677.98901 | - | - | - | - | - | - | 256683.6039 | 273713.3522 | 239526.8245 | 20652216.8 | 18284634.41 | 19315047.9 |
| Hexane, 2,2-dimethyl- | 5.19435 | 732 | 683.78218 | 20501160.56 | 22024680.47 | 19703376.25 | - | - | - | - | - | - | - | - | - |
| Di-tert-butyl peroxide | 6.68738 | 799 | 734.80833 | 33362621.03 | 38264455.84 | 42478928.18 | - | - | - | - | - | - | - | - | - |
| Spiro[2,4]hepta-4,6-diene | 7.23502 | 718 | 753.52472 | 14207688.15 | 16215287.06 | 14496692.21 | - | - | - | - | - | - | - | - | - |
| Hexanal | 8.1642 | 806 | 784.27904 | - | - | - | 10232505.44 | 10752441.28 | 9718010.626 | - | - | - | 15647206.2 | 16729449.74 | 18695666.65 |
| p-Xylene | 11.41653 | 907 | 867.67789 | 121720442.7 | 138719378.6 | 130371698.5 | - | - | - | 949488.93 | 991618.2977 | 1045889.765 | 18340241.74 | 21233880.32 | 23406367.33 |
| Styrene | 12.34228 | 883 | 889.88224 | 20305722.58 | 28070765.91 | 22082712.74 | - | - | - | 1161991.533 | 1117317.034 | 1192176.867 | 19228790.87 | 17061554.77 | 17965610.57 |
| Ethanone, 1-(2-furanyl)- | 12.7395 | 878 | 895.20097 | 83083459.13 | 121778481.3 | 110102123.7 | 5300619.743 | 5904903.935 | 6875700.819 | 1890797.794 | 1914655.107 | 2015001.425 | 59924786.87 | 60071844.39 | 51623207.05 |
| 2,4,4-Trimethyl-1-pentanol | 14.50282 | 911 | 923.04105 | 16835956.87 | 24825737.53 | 21833534.68 | - | - | - | - | - | - | - | - | - |
| Benzaldehyde | 14.91422 | 982 | 928.49285 | - | - | - | 53752377.25 | 52544428.81 | 53436585.23 | 19618935.08 | 19453466.87 | 20810044.89 | 354532484.9 | 339498521.1 | 365769957.9 |
| 2,4-Dihydroxy-2,5-dimethyl-3(2H)-furan-3-one | 15.97712 | 1173 | 942.57821 | 8692408.46 | 11361785.31 | 11484279.61 | - | - | - | - | - | - | - | - | - |
| 2-Octanol | 17.7717 | 979 | 966.35962 | 227804781.8 | 253928394.3 | 236033144.3 | 173187972.2 | 209284411.3 | 189328864.8 | 74268303.13 | 80174217.53 | 67681813.7 | 465542065.8 | 526594190.5 | 471002664.4 |
| D-Limonene | 17.79685 | 1018 | 966.6929 | - | - | - | 2601450.628 | 2493485.465 | 2729574.513 | - | - | - | - | - | - |
| Ether, 2-ethylhexyl tert-butyl | 19.22682 | 1141 | 985.64263 | 147413880.6 | 206311599.8 | 170028804.3 | - | - | - | - | - | - | - | - | - |
| Ethanone, 1-(1H-pyrrol-2-yl)- | 20.62881 | 1035 | 1015.68015 | 182743550.7 | 220941458.4 | 159712064.1 | 95802372.66 | 95479253.55 | 81171176.95 | 4142550.181 | 4780335.145 | 4227808.447 | 153827205.5 | 212862079.6 | 194384685.9 |
| Undecane | 22.37673 | 1115 | 1069.94343 | 5400984.353 | 7173328.04 | 7454060.551 | 2924515.961 | 3353334.976 | 3558815.964 | 1144498.28 | 1073139.485 | 1205016.965 | 39960488.9 | 37257143.29 | 37445868.12 |
| Nonanal | 22.64904 | 1104 | 1077.77469 | 11028315.4 | 13495441.31 | 11264857.08 | 21647685.77 | 21512437.28 | 18919482.14 | 546685.0031 | 614729.5277 | 584200.0738 | 32824252.47 | 27871355.86 | 31959928.7 |
| 4H-Pyran-4-one, 2,3-dihydro-3,5-dihydroxy-6-methyl- | 24.61798 | 1269 | 1122.69273 | 283781475.7 | 360958691.1 | 323148404.9 | - | - | - | - | - | - | 234690612.3 | 235633497.7 | 237161657.7 |
| Dodecane | 26.97086 | 1214 | 1164.99187 | 26313042 | 28600454.58 | 27805841.76 | 20882931.27 | 20738792.64 | 22388113.22 | - | - | - | - | - | - |
| 4-Methylpentyl 3-methylbutanoate | 28.49759 | 1154 | 1192.0624 | 19213906.44 | 20681151.25 | 19561724.44 | 5064134.464 | 5270616.066 | 5793915.179 | 2194379.283 | 2116260.317 | 2531543.004 | 35795247.77 | 37232666.9 | 42449007.59 |
| Tridecane | 33.11021 | 1313 | 1276.04871 | 14379650.86 | 13282426.81 | 13975269.74 | 10361262.39 | 10506830.33 | 10998119.66 | 2164987.594 | 1892769.343 | 2022108.444 | 40857659.76 | 36795999.07 | 42836090.57 |
| 4-Methylpentyl 4-methylpentanoate | 33.86962 | 1253 | 1290.24698 | 17119683.9 | 18203625.47 | 14569234.26 | 4999569.107 | 5463543.083 | 4350348.453 | 4335598.474 | 4073983.523 | 4329551.171 | 44168547.85 | 41052064.86 | 34136360.99 |
| Tricyclo[5.4.0.0(2,8)]undec-9-ene, 2,6,6,9-tetramethyl-, (1R,2S,7R,8R)- | 36.51739 | 1403 | 1342.19608 | 54122589.09 | 66302080.72 | 55679817.5 | 5526267.594 | 5846008.436 | 5403035.344 | 3281734.685 | 3212415.931 | 3183841.012 | 53552414.28 | 46843973.72 | 48166399.69 |
| Tridecane, 2-methyl- | 36.36803 | 1349 | 1338.34561 | 99789997.24 | 117994042.1 | 112180961.7 | 23758941.14 | 23414407.37 | 24620526.09 | 8815841.238 | 7527219.586 | 8272481.753 | 127559814.9 | 121114135.6 | 126538935.7 |
| 1,2,4-Methenoazulene, decahydro-1,5,5,8a-tetramethyl-, [1S-(1.alpha.,2.alpha.,3a.beta.,4.alpha.,8a.beta.,9R*)]- | 35.7203 | 1184 | 1325.62584 | 28149944.89 | 38872980.33 | 27806399.53 | 1653466.276 | 1561679.465 | 1658398.166 | - | - | - | - | - | - |
| 2-Butanone, 4-(2,6,6-trimethyl-1-cyclohexen-1-yl)- | 40.64389 | 1449 | 1424.05902 | 3535735.636 | 4578864.071 | 3584437.672 | - | - | - | 429543.4534 | 552312.1745 | 505842.7463 | - | - | - |
| n-Pentadecanol | 40.88291 | 1755 | 1429.124 | 22198553.4 | 21703957.25 | 25233206.94 | - | - | - | - | - | - | 56637022.33 | 48675708.1 | 52116477.49 |
| 1H-Benzocycloheptene, 2,4a,5,6,7,8,9,9a-octahydro-3,5,5-trimethyl-9-methylene-, (4aS-cis)- | 40.72089 | 1494 | 1426.11387 | 40098938.06 | 41128101.25 | 43759210.8 | 24833487.33 | 25013068.6 | 22685607.06 | 17166659.09 | 17875870.87 | 16869928.79 | 72228737.6 | 62895445.88 | 65847877.02 |
| Tetradecane, 2-methyl- | 40.94201 | 1448 | 1430.53434 | 22846539.64 | 26076595.29 | 27409309.67 | 9232541.911 | 9525737.966 | 10464569.32 | 7720645.563 | 7250349.89 | 7475269.395 | - | - | - |
| 5-Hydroxy-6-methoxy-8-[(4-amino-1-methylbutyl)amino]quinoline trihydrobromide | 42.04256 | 1499 | 1453.69777 | 67197389.3 | 71081693 | 74890943.42 | 19002859.64 | 18691196.77 | 17396709.64 | 13950918.87 | 13157497.91 | 12593749.38 | 94986508.14 | 83968780.67 | 80103021.59 |
| 3-Buten-2-one, 4-(2,6,6-trimethyl-1-cyclohexen-1-yl)- | 41.35655 | 1457 | 1439.16087 | 13244673.55 | 12080772.67 | 14627179.48 | 12967459.28 | 12806087.34 | 11568138.48 | - | - | - | - | - | - |
| .beta.-Longipinene | 42.41696 | 1398 | 1461.63152 | 29039911.93 | 28048388.03 | 29882934.28 | - | - | - | - | - | - | - | - | - |
| 4-Methylpentyl 8-methylnon-6-enoate | 48.56454 | 1659 | 1683.31828 | - | - | - | - | - | - | 1195120.659 | 1095513.7 | 1021923.169 | 10721711.84 | 9888304.737 | 8806478.773 |
| 2-Pentadecanone, 6,10,14-trimethyl- | 50.58652 | 1754 | 1845.47706 | 19192234.63 | 21317594.65 | 23844835.08 | - | - | - | 455263.198 | 503448.6603 | 374205.1015 | - | - | - |
| 1,2-Benzenedicarboxylic acid, bis(2-methylpropyl) ester | 50.92311 | 1908 | 1875.43098 | 15430829.42 | 14904329.43 | 17753327.36 | - | - | - | - | - | - | - | - | - |
| Heneicosane | 49.89695 | 2109 | 1790.37715 | 7546967.538 | 8680498.682 | 9272252.381 | - | - | - | - | - | - | 17385717.42 | 14671462.27 | 15703077.39 |
| Hexadecanoic acid, methyl ester | 51.16783 | 1878 | 1898.0412 | - | - | - | 8075917.203 | 9790031.651 | 10242203.86 | 4260514.804 | 5295474.557 | 5153469.331 | 3497338.046 | 3578519.09 | 4140877.915 |
| 1,2-Ethanediol, monoacetate | 6.07463 | 829 | 713.86686 | - | - | - | 66119204.91 | 65835356.64 | 65766837.87 | - | - | - | - | - | - |
| Heptanal | 10.67794 | 905 | 849.96269 | - | - | - | 1128303.339 | 1178840.593 | 1071725.864 | - | - | - | - | - | - |
| 1-Octen-3-ol | 14.17962 | 969 | 918.75796 | - | - | - | 2738735.989 | 2752231.832 | 2398630.439 | - | - | - | - | - | - |
| Orcinol | 17.35849 | 1235 | 960.88389 | - | - | - | 4194866.463 | 4276883.337 | 3729915.012 | - | - | - | - | - | - |
| Benzyl alcohol | 18.90626 | 1036 | 981.39462 | - | - | - | 46331286.49 | 44492942.33 | 48001731.71 | 5230611.57 | 5046914.406 | 5458170.949 | - | - | - |
| Benzeneacetaldehyde | 19.39964 | 1081 | 987.93285 | - | - | - | 19290948.22 | 20487598.17 | 17455909.75 | - | - | - | 88021845.56 | 84943359.39 | 100099132.3 |
| Benzaldehyde, 2,5-dimethyl- | 26.09279 | 1208 | 1149.59377 | - | - | - | 10260202.68 | 12601725.32 | 9008801.527 | - | - | - | - | - | - |
| Benzaldehyde, 2,4-dimethyl- | 26.48637 | 1208 | 1156.49574 | - | - | - | - | - | - | 366613.9539 | 409305.559 | 282340.5584 | - | - | - |
| Methyl salicylate | 27.56452 | 1281 | 1175.40256 | - | - | - | 1508352.772 | 2009814.94 | 2064171.5 | 723514.1893 | 852398.6602 | 841107.5659 | 34704268.95 | 30735677.08 | 33801057.62 |
| 1-Cyclohexene-1-carboxaldehyde, 2,6,6-trimethyl- | 28.95492 | 1204 | 1200.29278 | - | - | - | 6350289.643 | 6079863.101 | 5430381.211 | 496235.5905 | 504703.1292 | 520676.87 | 14268076.54 | 15775536.12 | 17741471.16 |
| Nonyl tetradecyl ether | 33.78423 | 2383 | 1288.42082 | - | - | - | 4312384.655 | 5098970.982 | 5725133.557 | - | - | - | - | - | - |
| Tetradecane | 38.12692 | 1413 | 1372.87489 | - | - | - | 9053350.428 | 9904710.101 | 10596130.4 | 1774317.412 | 1711996.444 | 1907938.816 | 47846943.27 | 40865466.42 | 41956590.4 |
| 1H-Benzocycloheptene, 2,4a,5,6,7,8-hexahydro-3,5,5,9-tetramethyl-, (R)- | 42.53949 | 1528 | 1464.22806 | - | - | - | 13065427.53 | 12935002.86 | 13617392.62 | 15770580.69 | 14394311.58 | 14122163.95 | - | - | - |
| 2(4H)-Benzofuranone, 5,6,7,7a-tetrahydro-4,4,7a-trimethyl-, (R)- | 43.71036 | 1426 | 1494.97822 | - | - | - | 9038658.967 | 9737730.325 | 9563427.228 | - | - | - | 31551077.29 | 28643659.39 | 31743919.67 |
| Methyl tetradecanoate | 48.81004 | 1680 | 1702.89264 | - | - | - | 3308796.321 | 3205878.846 | 3809931.86 | 793950.7209 | 1030607.667 | 962034.6868 | 32821379.73 | 47616212.04 | 35070974.19 |
| 9,12-Octadecadienoic acid (Z,Z)-, methyl ester | 53.06504 | 2093 | 2078.58595 | - | - | - | 1732976.704 | 2355406.099 | 1616145.911 | 871137.7524 | 1162484.576 | 818729.363 | 74646748.1 | 107724792.1 | 85520410.69 |
| 2-Propanone, 1-hydroxy- | 3.85872 | 698 | 638.13511 | - | - | - | - | - | - | 647106.1129 | 580690.1586 | 644276.2288 | - | - | - |
| Ammonium acetate | 4.40289 | 630 | 656.73303 | - | - | - | - | - | - | 3055283.444 | 3681361.439 | 3631453.374 | - | - | - |
| Acetic acid | 4.51609 | 576 | 660.60184 | - | - | - | - | - | - | 6939144.93 | 6408202.715 | 6804712.138 | 190109677.2 | 162360502.1 | 165212212.5 |
| 2,3-Butanediol | 8.36684 | 743 | 792.20637 | - | - | - | - | - | - | 1021499.279 | 1057824.878 | 1102521.797 | - | - | - |
| Benzaldehyde dimethyl acetal | 23.81563 | 1080 | 1109.66039 | - | - | - | - | - | - | 2139627.345 | 2092996.682 | 2297229.569 | 121842077.7 | 142608336.7 | 150679579.8 |
| Undecane, 3-methyl- | 27.07709 | 1150 | 1166.85486 | - | - | - | - | - | - | 333647.9107 | 355447.4192 | 380541.9049 | - | - | - |
| Nonanoic acid, methyl ester | 28.86417 | 1183 | 1198.50972 | - | - | - | - | - | - | - | - | - | 15876595.99 | 17885821.94 | 19112628.62 |
| 4-Methylpentyl 2-methylbutanoate | 28.76471 | 1154 | 1196.44965 | - | - | - | - | - | - | 8308444.794 | 8694386.194 | 9223911.239 | 145440527.2 | 126395527.9 | 127591693.2 |
| 6-Octenoic acid, 3,7-dimethyl-, methyl ester | 32.60216 | 1203 | 1266.72309 | - | - | - | - | - | - | 4672633.764 | 4720534.865 | 4759649.653 | 64736009.35 | 83296959.78 | 76356429.61 |
| 8-Methylnonanoic acid, methyl ester | 33.45401 | 1218 | 1282.35946 | - | - | - | - | - | - | 1700439.998 | 1665239.681 | 1686473.746 | 20450918 | 26850885.43 | 20653396.38 |
| Butanoic acid, 2-methyl-, heptyl ester | 34.46591 | 1317 | 1300.9985 | - | - | - | - | - | - | 376818.9043 | 323260.1509 | 283910.4524 | - | - | - |
| Tetradecane, 4-methyl- | 37.62455 | 1448 | 1363.01181 | - | - | - | - | - | - | 768315.0799 | 735986.9537 | 823959.7093 | 21844961.99 | 21507674.22 | 24900324.7 |
| Ylangene | 37.78017 | 1221 | 1366.06715 | - | - | - | - | - | - | 4988454.292 | 4775682.625 | 5013873.841 | - | - | - |
| Longifolene | 39.4697 | 1398 | 1399.31086 | - | - | - | - | - | - | 942190.6937 | 1250807.714 | 1147418.31 | - | - | - |
| (1R,2S,6S,7S,8S)-8-Isopropyl-1-methyl-3-methylenetricyclo[4.4.0.02,7]decane-rel- | 40.22096 | 1216 | 1415.09685 | - | - | - | - | - | - | 774604.7803 | 932870.2274 | 717319.2208 | - | - | - |
| Cyclopentadecane | 41.49248 | 1799 | 1442.04118 | - | - | - | - | - | - | 7581916.992 | 7840965.487 | 7349512.965 | - | - | - |
| 10s,11s-Himachala-3(12),4-diene | 43.88191 | 1494 | 1493.57038 | - | - | - | - | - | - | 169417912.8 | 162174606.2 | 166184291.8 | - | - | - |
| Pentadecane | 43.89061 | 1512 | 1492.8592 | - | - | - | - | - | - | 1865995.638 | 1696487.262 | 1543944.838 | 10482510.1 | 14526874.83 | 12613840.72 |
| 1H-3a,7-Methanoazulene, octahydro-3,8,8-trimethyl-6-methylene-, [3R-(3.alpha.,3a.beta.,7.beta.,8a.alpha.)]- | 44.91934 | 1398 | 1522.48746 | - | - | - | - | - | - | 547799.6944 | 474487.4363 | 510714.8767 | - | - | - |
| Pentadecane, 2-methyl- | 46.45881 | 1548 | 1572.53234 | - | - | - | - | - | - | 2237741.711 | 2185553.593 | 2379381.842 | - | - | - |
| Pentadecane, 3-methyl- | 46.63856 | 1548 | 1578.37562 | - | - | - | - | - | - | 527838.7523 | 569823.5697 | 509191.7615 | - | - | - |
| Isopentyl 8-methylnon-6-enoate | 47.08801 | 1559 | 1592.98608 | - | - | - | - | - | - | 1078222.951 | 1041364.982 | 1141287.024 | 8736412.069 | 9161147.719 | 10496551.05 |
| Hexadecane | 47.23198 | 1612 | 1597.66648 | - | - | - | - | - | - | 1245929.758 | 1131841.125 | 1245435.35 | 8344696.678 | 10394990.76 | 9604745.662 |
| Oxacyclotetradecan-2-one | 47.86852 | 1904 | 1637.32194 | - | - | - | - | - | - | 409955.9047 | 381566.7332 | 359742.1483 | - | - | - |
| 1H-Benzocyclohepten-9-ol, 2,4a-.beta.,5,6,7,8,9,9a-.beta.-octahydro-3,5,5,9-.beta.-tetramethyl- | 48.20806 | 1639 | 1659.76004 | - | - | - | - | - | - | 255054.4293 | 288328.2239 | 272199.6572 | - | - | - |
| Hexadecane, 2-methyl- | 48.31868 | 1647 | 1667.07034 | - | - | - | - | - | - | 1633921.223 | 1632706.79 | 1229544.052 | - | - | - |
| n-Nonadecanol-1 | 48.56091 | 2153 | 1683.07817 | - | - | - | - | - | - | 499391.9542 | 564755.3689 | 581820.4436 | - | - | - |
| Heptadecane | 48.82393 | 1711 | 1700.7292 | - | - | - | - | - | - | 2636733.157 | 2747639.839 | 2713617.431 | - | - | - |
| Pentadecanoic acid, methyl ester | 50.16001 | 1779 | 1807.89848 | - | - | - | - | - | - | 1286175.6 | 1105523.12 | 1242825.001 | 35469815.83 | 41786164.36 | 41952093.96 |
| Methyl hexadec-9-enoate | 51.33221 | 1886 | 1912.6826 | - | - | - | - | - | - | 523026.1974 | 639917.5654 | 609886.3009 | 6368679.942 | 9005081.616 | 8315809.217 |
| 1,2-Benzenedicarboxylic acid, butyl 2-methylpropyl ester | 52.17581 | 1973 | 1991.80519 | - | - | - | - | - | - | 432831.2875 | 614109.1232 | 456671.37 | - | - | - |
| Hexadecanoic acid, ethyl ester | 52.34196 | 1978 | 2007.71367 | - | - | - | - | - | - | 1306720.653 | 1196379.459 | 1247430.919 | 6840526.529 | 6398143.625 | 7186536.55 |
| Methyl stearate | 53.72181 | 2077 | 2143.54415 | - | - | - | - | - | - | - | - | - | 8787668.948 | 10318093.77 | 11378703.56 |
| Octadecanoic acid, ethyl ester | 54.43269 | 2177 | 2214.71035 | - | - | - | - | - | - | 467755.1655 | 430351.6624 | 480736.4766 | - | - | - |
| Hexanoic acid, methyl ester | 14.01881 | 884 | 916.62697 | - | - | - | - | - | - | - | - | - | 15858132.88 | 18177567.48 | 20506299.61 |
| 1,2-Propanediol, 1-phenyl- | 19.46193 | 1317 | 988.75819 | - | - | - | - | - | - | - | - | - | 227274462.8 | 231049297.9 | 274870149.8 |
| 1-Tetradecanol | 36.04399 | 1656 | 1333.48832 | - | - | - | - | - | - | - | - | - | 7433776.718 | 7414420.054 | 8118397.355 |
| 1,2,4-Metheno-1H-indene, octahydro-1,7a-dimethyl-5-(1-methylethyl)-, [1S-(1.alpha.,2.alpha.,3a.beta.,4.alpha.,5.alpha.,7a.beta.,8S*)]- | 37.68338 | 1125 | 1364.16686 | - | - | - | - | - | - | - | - | - | 12743455.1 | 14003908.16 | 16024959.74 |
| Cyclohexane, 1-ethenyl-1-methyl-2,4-bis(1-methylethenyl)-, [1S-(1.alpha.,2.beta.,4.beta.)]- | 38.73216 | 1398 | 1384.75754 | - | - | - | - | - | - | - | - | - | 36260887.61 | 36757248.5 | 32749337.55 |
| 3-Buten-2-one, 4-(2,6,6-trimethyl-2-cyclohexen-1-yl)- | 40.45827 | 1429 | 1420.12552 | - | - | - | - | - | - | - | - | - | 6942560.901 | 6265284.926 | 7186436.499 |
| 3-Buten-2-ol, 4-(2,6,6-trimethyl-1-cyclohexen-1-yl)- | 40.96086 | 1484 | 1430.77579 | - | - | - | - | - | - | - | - | - | 9546761.506 | 8149456.48 | 8584688.137 |
| Caryophyllene | 41.81482 | 1494 | 1448.87189 | - | - | - | - | - | - | - | - | - | 14773637.6 | 13016734.68 | 14690051.55 |
| Naphthalene, decahydro-4a-methyl-1-methylene-7-(1-methylethylidene)-, (4aR-trans)- | 43.24007 | 1502 | 1479.07381 | - | - | - | - | - | - | - | - | - | 12542818.79 | 11475828.16 | 12292362.1 |
| Naphthalene, 1,2,3,5,6,7,8,8a-octahydro-1,8a-dimethyl-7-(1-methylethenyl)-, [1S-(1.alpha.,7.alpha.,8a.alpha.)]- | 43.49928 | 1474 | 1484.56681 | - | - | - | - | - | - | - | - | - | 32210983.53 | 27410195.27 | 30574445.77 |
| Dodecanoic acid, methyl ester | 44.9952 | 1481 | 1524.95367 | - | - | - | - | - | - | - | - | - | 10972582.26 | 16272314.61 | 13185141.43 |
| Pentadecane, 2,6,10,14-tetramethyl- | 46.36067 | 1653 | 1569.34206 | - | - | - | - | - | - | - | - | - | 12128994.96 | 10991780.69 | 11152192.88 |
| Pentadecane, 8-hexyl- | 46.55043 | 2045 | 1575.51078 | - | - | - | - | - | - | - | - | - | 14060533.84 | 12178552.72 | 12140970.88 |
| Ethanol, 2-(9-octadecenyloxy)-, (Z)- | 49.02902 | 2336 | 1716.8492 | - | - | - | - | - | - | - | - | - | 9489641.922 | 9129118.912 | 7416583.546 |
| 9-Hexadecenoic acid, methyl ester, (Z)- | 50.96324 | 1886 | 1879.00197 | - | - | - | - | - | - | - | - | - | 7102708.738 | 7447323.333 | 8247627.021 |
