# Supplementary material for: Quantitative evaluation of quality indicators and flavor characteristics of the same variety of chili from different producing areas of China: An integrated multivariate analysis
Source: Food Chem X. 2026 Oct 7;39:104589. doi: 10.1016/j.fochx.2026.104589 (PMC13677961; doi:10.1016/j.fochx.2026.104589)
Supplement: Supplementary file 2 [file mmc3.docx]

**1** **Selected chromatograms of samples**

**1.1 B2-1**

**(1) Total ion chromatogram (TIC) in positive ion mode**

**(2) Base peak chromatogram (BPC) in positive ion mode**

**(3) Total ion chromatogram (TIC) in negative ion mode**

**(4) Base peak chromatogram (BPC) in negative ion mode**

**1.2 B6-1**

**(1) Total ion chromatogram (TIC) in positive ion mode**

**(2) Base peak chromatogram (BPC) in positive ion mode**

**(3) Total ion chromatogram (TIC) in negative ion mode**

**(4) Base peak chromatogram (BPC) in negative ion mode**

**1.3 B7-1**

**(1) Total ion chromatogram (TIC) in positive ion mode**

**(2) Base peak chromatogram (BPC) in positive ion mode**

**(3) Total ion chromatogram (TIC) in negative ion mode**

**(4) Base peak chromatogram (BPC) in negative ion mode**

**1.4 B8-1**

**(1) Total ion chromatogram (TIC) in positive ion mode**

**(2) Base peak chromatogram (BPC) in positive ion mode**

**(3) Total ion chromatogram (TIC) in negative ion mode**

**(4) Base peak chromatogram (BPC) in negative ion mode**

**2 Quality control (QC) chromatogram**

**2.1 Total ion chromatogram (TIC) in positive ion mode**

**2.2 Base peak chromatogram (BPC) in positive ion mode**

**2.3 Total ion chromatogram (TIC) in negative ion mode**

**2.4 Base peak chromatogram (BPC) in negative ion mode**
